# Leveraging Blockchain Technology for Secure Energy Trading and Least-Cost Evaluation of Decentralized Contributions to Electrification in Sub-Saharan Africa

**DOI:** 10.3390/e22020226

**Published:** 2020-02-17

**Authors:** Omaji Samuel, Ahmad Almogren, Atia Javaid, Mansour Zuair, Ibrar Ullah, Nadeem Javaid

**Affiliations:** 1 Department of Computer Science, COMSATS University Islamabad, Islamabad 44000, Pakistan; omajiman1@gmail.com (O.S.); atiajavaid477@gmail.com (A.J.); nadeemjavaidqau@gmail.com (N.J.); 2 Computer Science Department, College of Computer and Information Sciences, King Saud University, Riyadh 11633, Saudi Arabia; 3 Computer Engineering Department, College of Computer and Information Sciences, King Saud University, Riyadh 11543, Saudi Arabia; zuair@ksu.edu.sa; 4 Department of Electrical Engineering, University of Engineering and Technology Peshawar, Bannu 28100, Pakistan; ibrarullah@uetpeshawar.edu.pk

**Keywords:** blockchain, cryptocurrency, energy trading, fill rate, incentive mechanism, levelized cost of energy, service level, self-consumption, self-sufficiency and working capital

## Abstract

The International Energy Agency has projected that the total energy demand for electricity in sub-Saharan Africa (SSA) is expected to rise by an average of 4% per year up to 2040. It implies that ~620 million people are living without electricity in SSA. Going with the 2030 vision of the United Nations that electricity should be accessible to all, it is important that new technology and methods are provided. In comparison to other nations worldwide, smart grid (SG) is an emerging technology in SSA. SG is an information technology-enhanced power grid, which provides a two-way communication network between energy producers and customers. Also, it includes renewable energy, smart meters, and smart devices that help to manage energy demands and reduce energy generation costs. However, SG is facing inherent difficulties, such as energy theft, lack of trust, security, and privacy issues. Therefore, this paper proposes a blockchain-based decentralized energy system (BDES) to accelerate rural and urban electrification by improving service delivery while minimizing the cost of generation and addressing historical antipathy and cybersecurity risk within SSA. Additionally, energy insufficiency and fixed pricing schemes may raise concerns in SG, such as the imbalance of order. The paper also introduces a blockchain-based energy trading system, which includes price negotiation and incentive mechanisms to address the imbalance of order. Moreover, existing models for energy planning do not consider the effect of fill rate (FR) and service level (SL). A blockchain levelized cost of energy (BLCOE) is proposed as the least-cost solution that measures the impact of energy reliability on generation cost using FR and SL. Simulation results are presented to show the performance of the proposed model and the least-cost option varies with relative energy generation cost of centralized, decentralized and BDES infrastructure. Case studies of Burkina Faso, Cote d’Ivoire, Gambia, Liberia, Mali, and Senegal illustrate situations that are more suitable for BDES. For other SSA countries, BDES can cost-effectively service a large population and regions. Additionally, BLCOE reduces energy costs by approximately 95% for battery and 75% for the solar modules. The future BLCOE varies across SSA on an average of about 0.049 $/kWh as compared to 0.15 $/kWh of an existing system in the literature.

## 1. Introduction

With the world population explosion and industrialization growth in developing countries, consumers’ need for energy consumption has reached an unprecedented level. As countries like India and China are speedily industrializing, the present limited set of traditional energy generating plants like natural gas and coal will no longer be sufficient [1]. Therefore, there is a need for the transition into renewable energy resources (RES) like micro-renewable, biomass, combined heat and power, geothermal, solar, and wind energy, which are decentralized [1]. A traditional energy system consists of energy generating plants, distribution and transmission lines, transformers, substations, and consumers. In a traditional energy system, centralized energy system (CES) plays a major role in coordinating, controlling, and managing different energy generating plants. However, the CES becomes inefficient because of high operation and maintenance cost (OM), lack of trust and untimely response (due to high traffic of energy requests). CES generates electricity at large scale and ensures economies of scale by reducing the energy set up cost; however, this benefit is often not felt in rural communities [2]. It is, therefore, necessary to consider the condition under which the energy needs of rural communities can be satisfied more efficiently via decentralized manners [3]. Decentralized energy system (DES) provides independent and flexible sources of energy that satisfy several communities. DES is a network of distributed RES off the grid, where each RES has a controller that communicates and negotiates conveniently with others to achieve their goals without the influence of a central controller. DES provides energy supply to critical facilities during emergency and outage, reduces energy storage cost, ensures fault tolerance and local accountability. However, frequent intermittency of RES’s outputs at participants level can make DES complex [4,5].

Access to cheap electricity via the solar home system (SHS) is an evolving paradigm that integrates RES. SHS is a small-scale power system that offers a cost-effective way of satisfying household energy demand in the absence of the main grid [6]. greenSHS can improve the lives of approximately 1.1 billion people with inaccessible electricity worldwide, predominantly affected regions include sub-Sahara Africa (SSA) (i.e., 620 million), the geographical focus of this study; greenAsia (350 million); and the Caribbean and Latin America (20 million) [7]. In SSA, firms and households can withstand several hours of brownouts, which limit their potential usage of electricity. In Liberia, more than half of the households connected to the grid have no supply of electricity. Also, countries like Sierra Leone and Uganda experience energy reliability issues. In addition, more than 50% households in Nigeria, Ghana, Zimbabwe, and Burundi have no access to electricity [8]. The cost of energy in SSA is highest in the world, where regulatory tariff is below the cost-recovery levels. It potentially increases the existing problem of reliability of energy. To address these problems, this paper proposes a blockchain-based DES (BDES) to increase energy efficiency and fix tariff regulatory issues through a proposed price negotiation mechanism [9]. Reliable energy can contribute in increasing the uptake across and within countries, and countries can increase their tax revenues by more than 4% per year to address the problem of reliable energy [8]. Because of the energy market size of each country in SSA, there is a need for a pool of energy resources, which can reduce energy supply risk by taking advantage of economies of scale. In this study, a blockchain-based energy trading system is proposed, which automates and minimizes the cost of maintaining unpredictable growth in the number of participants in the energy market. Energy security has been debated over the past two decades as vital agendas for increasing country’s uptake [10]. These energy security policies are mostly enforced in countries, such as the US, China, and the Middle East, rather than in the SSA. Large-scale resource mining, such as uranium, has been the focus of energy security in SSA, which has drawn investment boom; however, RES is given little attention. Nonetheless, Africans have tried to increase their local energy security by using variety of energy resources; however, there are no reliable data records for energy security and energy consumption by customers. Other factors that limit SSA energy security include energy supply risk, which can be mitigated by effective policy-making and regulation using cost-effective technology, and technical challenges like energy theft, which pose serious problems in energy management, especially in Botswana [11]. The regulatory framework also poses serious organizational and record-keeping challenges to energy security in SSA. Non-technical challenges include the problem of data access and ownership of large amounts of data generated from the energy consumption of consumers, which is personal and high value. Customers, however, are interested in the protection of their data, while utilities, on the other hand, depend on the data of customers to efficiently operate their energy plants. However, policy-makers are also interested in the data from consumers for cost-effective energy planning policies. Therefore, protecting the privacy of consumers’ data and providing each stakeholder with the level of access it needs, is important. Data privacy is another problem in SSA, which poses a vital question of how secure consumers’ data is to an adversary, and therefore an efficient mechanism needs to be provided. Also, due to the infrastructural strength of the energy system in SSA, one key challenge is communication, which comes with cybersecurity concern on the invasion of consumers’ private data. This challenge arises when digital electricity infrastructure is implemented. Based on the above-mentioned challenges, high cost of energy generation in SSA, energy policy, and regional electrification investment plans may raise research questions about.

- Is there any feasible long-term cost-effective energy planning model for energy generation in SSA?
- Are there any solutions to historical antipathy or lack of trust and security that can jeopardize the process of regional decentralized energy cooperation in SSA?
- Are there any technologies that provide a secure trade arrangement for cross-border electricity, which can effectively solve the problem of fair energy trading and payment in SSA?

A cost-effective energy planning model is used to compare the energy generation capacity of a CES with the options of DES that often consider solar with battery storage [2]. brown Several energy planning models were proposed in the literature to provide cost-effective solutions to classes of DES for different countries [12,13,14,15,16,17,18]; however, their focus was on isolated geographical locations. Although, the authors of [19] have proposed a model for energy planning for multiple SSA locations. However, its implementation was precluded by the lack of available knowledge of security, privacy, trust and energy trading. The authors of [11] highlighted the challenges of introducing smart grid (SG) in SSA to tackle energy crises, such as cybersecurity, regulatory framework, technical and non-technical issues. However, they do not consider energy crises in terms of declining storage capacity and electricity theft. Also, SSA’s critical energy infrastructures are weak and unreliable in terms of energy storage and management in contrast with developed countries. Energy generations in SSA are insufficient to satisfy the increasing demand of the region, especially in peak time demands [11]. Therefore, it is necessary to introduce technology that can pool energy resources together. Although, the authors of [20] argued that introducing SG can provide equity and access to electricity in SSA. However, other SG challenges are not considered, such as lack of trust, privacy, and security. Based on the above limitations, it is clear that blockchain technology is not explored in SSA, which is the motivation of this study. In this paper, the main objective is to propose a secure BDES framework that leverage blockchain technology for decentralized electrification in SSA. First, a cost-effective energy planning solution is designed to estimate the cost of a CES, which is comparable to the cost of a DES, and analysis of the cost–benefit trade-off between energy costs of CES, DES, and the proposed BDES is also considered. Second, the issues of how to increase local energy supply through self-consumption and self-sufficiency or a secure cross-border exchange of energy via blockchain-based trading are discussed in this paper. Third, this study also proposes a blockchain-based energy price negotiation and incentive models that encourage prosumers to contribute their excess energy generations to the market. Fourth, excess rewards or incentives of prosumers are traded within the blockchain network using the proposed cryptocurrency trading framework. Furthermore, simulation results aim to illustrate the potential benefits of blockchain technology in a DES. Besides, it also provides high-reliability service at system protection and cost that outperforms the existing highly undependable grid system. At the end of the paper, all abbreviations used in this work are presented.

This paper is the extension of the previous work [21] that provides the proposed BDES under study. The impact of energy reliability on generation costs based on the fill rate (FR) and service level (SL) for decentralized SHS using daily variations of location-specific solar resource data with $1^{{}^{\circ}}$ latitude and longitude was proposed in the previous work. A Quasi–Newton’s method was used to optimize solar modules and battery storage costs, whereas a multi-step optimization method was used to minimize the least-cost system. In the current study, the contributions are listed below.

1. This paper proposes a secure BDES that addresses the issue of historical antipathy (i.e., lack of trust) and security in order to establish decentralized electrification in SSA.
2. A blockchain-based mechanism for the trading and payment of energy in SSA is proposed to ensure fair regional and cross-border energy trade. New methods of self-consumption and self-sufficiency are included in the proposed energy trading mechanism to enhance energy trading for regional energy producers.
3. A cost-effective energy planning model is proposed to quantify the impact of energy reliability on generation costs. The proposed energy planning model is used to compare the economic energy generation costs between CES and DES in SSA.
4. Energy trading price negotiation and incentive mechanisms are also proposed to encourage more participation of energy users in energy trading and payment. Also, a mechanism for cryptocurrency trading is introduced to reduce excess rewards and alleviates the electricity debt of customers.

The organization of this paper is as follows. Section 2 provides the literature review, and Section 3 describes the proposed framework and problem formulations. Section 4 presents the simulation results and their discussions, and Section 5 provides the research implications. Finally, the conclusion and future work direction are given in Section 6.

## 2. Literature Review

This section presents the review of related work based on energy management in SG, blockchain for SG and least-cost evaluation of decentralized electrification in SG.

### 2.1. Energy Management in SG

In SG, prosumers are entities that produce and consume energy. Small-scale prosumers are individual prosumer who do not generate energy for commercial purposes, however, generate energy for its needs. Note that solar system is more suitable for small-scale deployment. Therefore, small-scale prosumers’ energy can be metered by larger suppliers [22]. In addition, small-scale prosumers are not able to participate in the wholesale energy market because they contribute little to generation, transmission, and settlement procedures. Whereas, large-scale prosumers are regional utilities with small-scale energy deployment [23]. If prosumers are unable to meet their current energy demands, then the required energy demands are fulfilled from the grid. To ensure the stability of the grid and reduce peak requests, the utility employs demand-side management (DSM). It encourages customers to optimize their energy use through direct control or incentive mechanisms like dynamic pricing schemes [24]. DSM plays a vital role in shaping the overall energy demands of consumers along with distributed energy resources (DERs). DERs are small-scale RES generated at the consumers’ side. DSM further reduces investment cost and improves the efficiency of the energy system. In addition, a dynamic pricing scheme like time of use is employed to incentivize consumers in terms of reduced cost to shift their high load demands from on-peak to off-peak time slots [25]. However, consumers can create rebound peaks during off-peak time slots as they exploit the low price in these time slots despite efficient scheduling mechanism. Although, DER owned by a single prosumer can be optimally operated and controlled [26]. However, operation and control of DERs from different prosumers may raise problems in storage and generation. In the literature, the game theory approach is used to model and coordinate individual prosumer within peer-to-peer (P2P) environment such that each entity hopes to maximize its payoff [27,28,29,30]. In P2P energy trading, fixed price mechanism becomes inefficient as the participants of energy trading need to set energy price based on negotiation, auction and sometimes through an analytical approach. Also, the accepted price by participants is usually lower than the grid price. Nevertheless, these participants create peaks in demand as they take advantage of the low price. In contrast to the fixed price mechanism, in a competitive market, the energy dual price variables (i.e., selling and buying) can be optimized [22]. However, the optimized price variables cannot be used to provide incentives if one participant has asymmetric information.

### 2.2. Blockchain for SG

Different countries have started sandbox implementation of blockchain projects in the energy sector by relaxing energy regulations at a minimal scale to allow innovations. This approach encourages energy inventors to test their prototype systems without affecting the energy system. Singapore National Electricity Regulations and the United Kingdom have deployed this approach, which attracted prominent blockchain investors to pilot their ideas with jurisdiction [31]. The government of New York State encourages investors to pursuit prototype projects by applying a range of technologies, not necessarily blockchain, with less restrictive regulations. Two years ago, utility services of network companies, such as Tennet Holding BV and Vattenfall AB, have adopted a testing software project for blockchain, with 23 utility companies like Iberdrola SA and Total SA participating in the project [32]. These utility companies participated because they wanted to test how blockchain works and whether it will pay off when used for trading [32]. Similar projects have been developed like Brooklyn that support efficient operations of energy systems, reduce energy cost, improve resilience and reliability [31,33]. Blockchain may assist in solving several problems of optimization [34] and energy reliability by providing visibility and control of real-time power injection and flow from DERs at substation level [35]. Large penetration of DERs without the precise cybersecurity measures, such as monitoring and trustworthy communication, may jeopardize the energy system and cause outages and reliability problem for consumers [35]. Attackers may modify DERs data being sent to energy management system by exploiting the insecure communication channel to compromise DER’s control algorithm. Blockchain can be used to mitigate this type of attack by recording the data transaction time. In addition, it authenticates and verifies transaction data to drop any command that contradicts commands written in the smart contract [31]. Blockchain increases reliability and trust among participants, which are important features in data management that are not part of the traditional system. On the other hand, the traditional system is vulnerable to a single point of failure. If data is stored at a single point, then it will be difficult to audit and track due to the time of request. Blockchain helps to ensure that data is stored across all nodes [36].

Blockchain technology provides security, and improves communication and trade by increasing transparency of transactions. Also, blockchain provides traceability of consumers’ payment data and trust between participants [37]. Furthermore, blockchain’s cryptocurrency provides an avenue for autonomous market entities to participate in the energy market using P2P market model (i.e., a model providing direct energy trading between peers [27]). Blockchain and P2P energy infrastructures are also necessary for developing countries to achieve new and low cost of RES [37]. P2P energy trading system consists of buyers and sellers of RES, where a fabricated price of energy autonomously depends on the number of participants (i.e., producers and consumers) [25]. However, the fabricated price of energy may result in imbalance of orders (i.e., imbalance of order is a situation resulting from excess of buy or sell orders on trade exchange [38]). Blockchain can significantly minimize the fabricated price with the help of a smart contract. Blockchain smart contract is a computer script that provides terms and conditions, binds participants, and also transfers digital currencies or assets between participants [37].

Based on trust, blockchain encourages rural settlers to produce and trade their energy with other participants in the same community. It also encourages the sale of surplus energy to the main grid via transmission lines. Consumers from another community can purchase energy via mobile devices as pay-as-you-go service or nonpayment as community services. Rural households can generate their energy using SHS in the case, where they cannot access electricity from the grid. Households with SHSs get incentives instantly when they generate enough energy to meet consumers’ demand through energy trading. Energy trading can be conveniently performed on blockchain network through digital tokens, and tokens can be redeemed for a remote cryptocurrency. The immutable feature of the blockchain makes energy trading more secure as an individual or group cannot alter trade data.

Nowadays, different developments and applications of blockchain have emerged [39]. Blockchain 1.0 is based on digital currency systems, such as Bitcoin. Blockchain 2.0 incorporates the smart contract and other digital assets, which support applications within the blockchain and the currency systems. Blockchain 3.0 combines infrastructures like the Internet and mobile communications [39]. Based on the different developments of blockchain, the future evolution of blockchain with energy trading can achieve interoperability, adaptability, privacy, sustainability, and instantaneous transactions [39]. Consumers can increase their market sizes, as documentations, payments, and energy exchange improve the efficiency of doing business. In addition, by eliminating the need for a third party to approve transactions between consumers, payments are made at a cost lower than the conventional utility rates.

### 2.3. Least-Cost Evaluation for Decentralized Electrification in SG

Energy reliability refers to the performance of a power system [40]. Energy reliability is evaluated based on the assumption of practical applications of probability theory. The assessment of energy reliability’s impact on cost is examined based on adequacy and security. Energy security refers to the ability of a power system to overcome dynamic and external disturbances, which include loss of generations, transmission, and voltage instability [40]. On the other hand, energy adequacy refers to the availability of facilities within the system that can fulfill the customers’ load demands and operational constraint of the system. However, energy adequacy does not address dynamic disturbances like voltage instability [40]. Note that the investment cost is used to relate reliability with economics. Based on this approach, levelized cost of energy (LCOE) is a method used to compare the economic cost of a RES relative to other RES. The economic costs include the cost of energy generating plant (may include fuel cost), maintenance cost, and other related taxes and subsidies that are based on the operation of energy generating set. Mathematically, LCOE is defined as the net current value in unit-cost of energy over the lifespan of the energy generating plants in cost per kilowatt-hour (kWh) [12].

This paper discusses some of the uses of LCOE in the electricity market for different countries. The LCOE of wave energy conversion is considered for four different potential sites along the US pacific coast during one year [12]. The proposed scheme is used to model the device power performance and estimates the capital expenditure and operation expenditure reduction by 75% to achieve LCOE of 0.30 $/kWh. The wave energy conversion and wave energy conversion array techniques achieve annual production of ~200% through control strategies. However, variability and intermittency issues associated to the performance of wave resources are not considered. In China [13], LCOE is used to estimate the on-shore wind power that will decline by 13.91% from 0.40 RMB/kWh in 2016 to 0.34 RMB/kWh in 2025. Although on-shore wind power achieves the grid parity with the carbon pricing of 10, 35, and 60 RMB/kWh for 2019, 2017, and 2016, respectively, by comparing the on-grid price of coal-fired power and LCOE. Furthermore, grid parity is delayed based on sensitivity analysis of high discount rates, curtailment rate, learning rate, and low capacity factor. Besides, they achieve grid parity with high carbon pricing. However, the variation in carbon tariff will increase the storage need. In Mauritius [14], off-shore wind and solar technologies are used with capital factor to determine the impacts of LCOE on bagasse generation, utility-scale PV, and landfill gas-to-energy, which are below average costs and fuel oil’s operating costs. The results enable policy-makers to prioritize cost-effective solutions. However, the performance of photovoltaic (PV) technology for short and long term range with reliable information is not provided by the study in Mauritius. In Pakistan [15], LCOE is used to determine the performance of wind farms in terms of the average capacity of 34.50%, real availability of 90% and 97% of technical performance. The costs of 0.113 $/kWh and 0.040 $/kWh for 1–10 and 11–20 years are estimated from LCOE. Based on the report, the low production cost is attained per kWh as it is also compared with other technologies.

In Italy [16], an opportunity LCOE is used to aid stakeholder in selecting corporate power purchase agreement price and contractual length. An opportunity LCOE is estimated such that the contractual length should be within 7 years, whereas corporate power purchase agreement price ranges from 75 *£*/MWh to 100 *£*/MWh. In Korea [17], real LCOE is responsible for direct and indirect generation costs and economic theory is used to derive these costs. Indirect cost is used to quantify the importance of the energy generation mix. The real LCOE provides accuracy of power generation costs and quantities of RES. The results show that for extra units of RES, indirect cost savings are achieved. The authors of [18] analyzed the levelized cost of system for six different island geographies by exploiting PV, wind, battery storage, and pumped hydro-storage, based on sizes and contexts. The levelized cost of the system shows the generation of electricity will increase considerably with the rise in RES penetration up to 40% to 80% optimally. Reports show that the PV has significantly contributed in halve of the islands. However, storage is the requirement for saving overproduction as the dependence upon diesel production is limited. The authors of [19] proposed LCOE for decentralized solar power systems in SSA. In their framework, a standalone SHS is considered with solar and battery capacities using fraction-of-demand-serve. Also, fraction-of-demand-serve is used to measure the reliability via a multi-step optimization to derive the least-cost system. However, the authors did not consider energy trading in a trusted and secure environment for the decentralized SHS.

## 3. Proposed Framework

The framework consists of main grid that is the major source of energy, whereas prosumers and consumers belong to the blockchain network. Prosumers use SHS to generate energy and engage in trading of energy and cryptocurrency with other prosumers and customers. Consumers receive energy directly from the grid and can also participate in the trading of energy and cryptocurrency with each other and prosumers. Note that blockchain ensures a trustful and secure environment for trading. In addition, it serves as a broker between all participants in the proposed framework. Furthermore, blockchain uses the excess coins or incentives from prosumers and consumers to settle their financial debts. The subsections below describe the components of the proposed framework.

**Figure 1:**
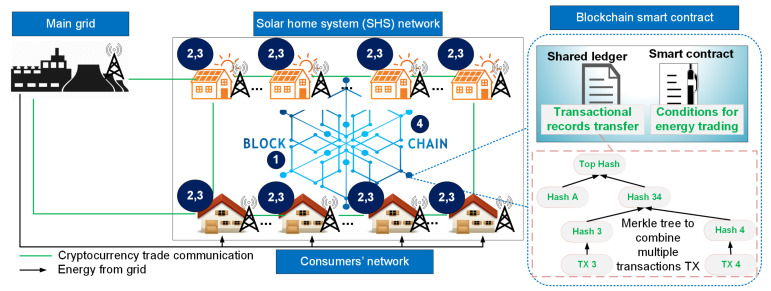
Proposed framework: 1: Financial mode; 2, 3: Metering mode and Trading platform; 4: Industrial standard.

A blockchain structure is chain-shaped [41,42,43]. Every data block contains transactions, which are replicated and accessible by all participants via a distributed network [44]. In Figure 1, blockchain has two parts: first, the shared ledger and second, the smart contract. The shared ledger contains the transaction records and the hash values, while the smart contract provides conditions for energy trading. The present hash value depends on the hash of previous block to form the Merkle structure. The initial block in the blockchain is known as the genesis block, and a newly created block is validated and sequentially chained to the genesis block or previous block to create a chain of blocks. The implementation of blockchain has some basic components [41].

- The blockchain user creates a new transaction, which is visible to other nodes in the network.
- Each node validates each transaction at a certain period. Invalid transactions are discarded. Contrarily, valid transactions are packed into a new block through the mining process.
- Before a block is committed to the blockchain, a notification is sent to all mining nodes within the blockchain network. Mining nodes, which receive the committed block, will verify the validity of the block with a hashing mechanism [45]. If the block passes the verification test by the majority of mining nodes, then it will be chained at the end of the current blockchain. Thus, a consensus is established. In this paper, proof of authority consensus mechanism is used [46]. The total usage of energy is measured and recorded with the help of smart meter and it indicates the first proof of their work for all prosumers.

### 3.1. Structure of the Blockchain

#### 3.1.1. Prosumers

Energy prosumers are consumers that are involved in energy generation. When they generate more energy than their demands, the surplus energy is sold to the other consumers. On the contrary, when prosumers are energy deficient, they purchase the required energy from local energy market by taking part in trading or from the grid. Small-scale prosumers are prosumers who cannot function economically without remuneration and self-consumption [47]. Solar energy is more suitable for small-scale deployment, whereas other RES, like wind turbine, is more useful on a large-scale deployment [47]. In this paper, prosumers have household standalone SHS for their energy generations. SHS consists of one or more PVs, charge controllers (to prevent batteries and appliances from damage), solar cells, and a battery to store the harvested energy.

#### 3.1.2. Energy Consumers

Energy consumers are entities, which consume electricity. Consumers pay their electricity bills through cash, debit cards at the start of each month, or in arrears by quarterly account. Consumers use the same price for energy consumed per kWh. Consumers who have dual meters pay less during night consumption and consumers who have a capped rate, pay more if they exceed a consumption threshold. Blockchain technology will be used to minimize the vetting processing time during electricity bill payment.

#### 3.1.3. Smart Contract

Blockchain’s smart contract is a computer script that provides terms and conditions, which binds participants and also transfers digital currencies or assets between participants. Smart contract provides enforcement, and minimizes contract signing and regulatory costs [48].

#### 3.1.4. Financial Mode

Financing distributed and decentralized SHS’s startups project is tedious due to inadequate financing and lack of trust between participants. Energy brokers with high credibility are unable to review the process of authenticating the system due to high cost of processing long-time contract rules [48]. The consensus mechanisms of blockchain establish trust and participants’ contract rules can be unanimously processed and validated. In addition, stored records of participants in blockchain simplify the business process, thereby improve the financial efficiency of decentralized SHS.

#### 3.1.5. Metering Mode

Smart meters installed in the residential homes of all prosumers and consumers provide the first proof of their work for energy consumed or sold. Without the blockchain, there is an issue of inaccurate metering of prosumers caused by substandard measuring devices, information losses during transmission, and lack of focus by the data entry staffs [48]. Blockchain provides a digital precision of the smart meters’ data and management. Access restriction and time of access are recorded and proper digital mapping is enforced to achieve accurate measurement through a well-defined rule.

#### 3.1.6. Trading Platform

Prosumers utilize their energy generation and can buy energy from other prosumers, if required. With blockchain, prosumers can sell their excess generations to neighboring prosumers or consumers via P2P trading mode. By trading, the excessive energy is not wasted. By contrast, in absence of trading, the excessive amount of energy would be wasted. Besides, prosumers can change their operating mode if their local energy generation is insufficient and then buy the needed energy from other prosumers. Thus, blockchain ensures energy is fully utilized and also minimizes energy wastage.

#### 3.1.7. Industrial Standard

Blockchain technology has a high degree of adopting decentralized SHS. Blockchain creates a trustless environment and establishes P2P trading market without the trust endorsement of a third party. Therefore, it reduces the difficulty of unifying industrial standards [48].

### 3.2. Problem Formulations

To avoid verbosity, only relevant formulations from the previous study [21] are presented.

### 3.3. The Isoreliability Curve

The set of solar and battery storage capacity, which satisfy the FR is calculated in an hourly time slot. The isoreliability is used to depict the constraint imposed by system [19]. The paper considers dynamics of the time slot, *h*; for solar, $C_{s}\left(h\right)$; and battery, $C_{b}\left(h\right)$, capacities (subscript *s* and *b* denote solar and battery), individually. After battery charging or discharging, the excess energy (or deficit) is calculated as

(1) $$\delta E_{b}^{ex}\left(h\right)=C_{s}\left(h\right)I_{s}\left(h\right)-L\left(h\right),$$

where $I_{s}\left(h\right)$ is the insolation, which is measured in kWh and L(h) is the load at *h*, measured in kWh. To determine amount of available energy, a state of charge SOC is used to define the energy stored in a battery at a given time slot *h* as [19]

(2) $$SOC\left(h+1\right)=\max(0,\min\left(C_{b}\left(h\right),SOC\left(h\right)+\delta E_{b}^{ex}\left(h\right)\right)).$$

At a given time slot *h*, the unmet load is calculated as [19]

(3) $$L^{unable}\left(h\right)=\max\left(SOC\left(h+1\right)-SOC\left(h\right)-\delta E_{b}^{ex}\left(h\right),0\right).$$

$FR_{N}$ is calculated as the fraction of demand, which is to be satisfied through the available energy without loss of energy. *N* is the total number of simulations. $FR_{N}$ represents the fraction of demand, where recovery is possible, and it is also measured empirically by averaging the number of correctly delivered energy to the number of demands. $FR_{N}$ depends on the service level $SL_{N}$. Besides, $SL_{N}$ is the expected probability of not hitting the stock-out. In addition, $SL_{N}$ represents the trade-off between the cost of storage and stock-out. $SL_{N}$ is calculated as

(4) $$\begin{array}{c} SL_{N}=\left\{\begin{array}{cc} 1, & if\;E_{p}^{val}\left(h\right)>E_{p}^{req}\left(h\right)\\ 0, & \mathrm{otherwise}, \end{array}\right. \end{array}$$

where $E_{p}^{val}\left(h\right)$ denotes the available energy during the time slot *h* with subscript *p* to demote prosumer, and it is given as [49]

(5) $$E_{p}^{val}\left(h\right)=\left(SOC\left(h\right)-SOC^{thr}\left(h\right)\right)E^{rt}\left(h\right),$$

where $SOC^{thr}\left(h\right)$ denotes the SOC’s threshold thr and $E^{rt}\left(h\right)$ denotes the rated capacity of the battery storage. $E_{p}^{req}\left(h\right)$ represents the required energy and it is calculated as [49]

(6) $$E_{p}^{req}\left(h\right)=\left(SOC^{\max}\left(h\right)-SOC\left(h\right)\right)E^{rt}\left(h\right),$$

where $SOC^{\max}\left(h\right)$ represents the maximum SOC at a given time slot *h*. The $FR_{N}$ is calculated as

(7) $$\begin{array}{c} FR_{N}=\left\{\begin{array}{cc} 0, & if\;SL_{N}=0\\ \max(0,d_{N}), & \mathrm{otherwise}, \end{array}\right. \end{array}$$

where $d_{N}$ is given as [19]:

(8) $$d_{N}=1-\frac{\sum_{i=1}^{N}L^{unable}\left(h\right)}{\sum_{i=1}^{N}L\left(h\right)}.$$

Isoreliability curve is generated by considering the dynamics of insolation, capacities of solar and battery as a function in their compact form. The storage and solar capacities are optimized using the Quasi–Newton method [50] to get two optimal points ($\bar{C_{h}^{s}},\bar{C_{h}^{b}})$. The isoreliability curve values used in this paper are taken from [19] for each degree of latitude and longitude across the SSA.

#### 3.3.1. BDES

The prices of solar and storage are formulated to derive their respective costs, which depend on the isoreliability curve values. A battery replacement cost is used to derive the total cost of storage. Thus, the capital cost of the battery is defined as [19]

(9) $$B_{c}=C_{b}Cost^{b},$$

where $Cost^{b}$ is the battery cost per kWh, which is computed as [19]

(10) $$Cost^{b}=\frac{1-{\left(1-r\right)}^{m}}{1-{\left(1-r\right)}^{LT}}Pr^{b},$$

where *r* is the discounted rate, *m* is the project term, $Pr^{b}$ is the total price of battery including replacement cost, and LT is the lifetime of the battery. To prolong the lifespan of solar, its solar derating is performed. Thus, the capital cost of solar is computed as [19]

(11) $$S_{c}=C_{s}\left(h\right)\left(\frac{Pr^{s}}{\alpha }+Chrg^{c}\right),$$

where $Pr^{s}$ is the solar module’s price per kW, $Chrg^{c}$ is the charge controller, and α is the derating factor taken to be 0.85 [19]. The derating factor is used to account for conversion losses, panel mismatch, dirt, and wiring issues. The total capital cost of the system $SYS_{c}$ is given in Equation (12), and it is calculated based on average daily load $Av\bar{g}DL$, capital cost per unit peak load $C_{L}^{peak}$, and system’s peak load capacity $S_{L}^{peak}$.

(12) $$SYS_{c}=\left(B_{c}+S_{c}\right)Av\bar{g}DL+C_{L}^{peak}S_{L}^{peak},$$

(13) $$CRF=\beta \times r\frac{{\left(1+r\right)}^{m}}{{\left(1+r\right)}^{m}-1}.$$

Based on Equation (13), CRF is the capital recovery factor, *r* is the annual discounted rate, and β is the blockchain incentive such that 0<β≤1. The BLCOE is calculated using Equation (14).

(14) $$BLCOE=\frac{SYS_{c}\times CRF+OM\times S_{L}^{peak}}{365\times Av\bar{g}DL\times FR_{N}}.$$

The objective is to minimize *BLCOE* such that $\left\{C^{s},C^{b}\right\}$ falls within the isoreliability curve. Thus, the total capital cost is formulated as [19]

(15) $$B_{c}+S^{c}=C_{s}\left(\frac{Pr^{s}}{\alpha }+Chrg^{c}\right)+C_{b}Pr^{b}.$$

Although, *r*, mean load, peak load, α, $Pr^{b}$, and $Pr^{s}$ are all parameters to be regulated separately of isoreliability curve. However, providing optimization of all parameters is like minimizing the total capital cost given in Equation (15), which is constrained by the isoreliability curve [19]. A continuously differentiable isoreliability can be discretized as analytical solution exists for the problem. Therefore, the integral search on the reliability curve is used to find the point of minimizing the cost.

#### 3.3.2. CES

In CES, two important component costs of satisfying energy demands via centralized generation are required: the variable cost $V_{c}$ of energy generation based on centralized technology, which is measured in $/kWh, and the infrastructure cost of transmission line $T_{c}$, which is measured in $/m/year [3]. The total levelized cost of centralized energy $C_{LCOE}$ is defined as

(16) $$C_{LCOE}\left(FR,V_{c}\right)=L_{c}\left(FR\right)T_{c}+E_{c}\left(FR\right)V_{c},$$

where subscript *c* denotes centralized and $E_{c}\left(FR\right)V_{c}$ is the total yearly energy consumption for households with grid connection. Let $L_{c}\left(FR\right)$ be the number iterations defined as a function of FR. Note that the transmission levelized cost is defined as [3]

(17) $$T_{c}=\frac{\mathrm{PV}\mathrm{generation}\mathrm{cost}}{\mathrm{Annualized}\mathrm{discounted}\mathrm{cost}\mathrm{of}\mathrm{operation}}.$$

$T_{c}$ is used to express the discounted cost of operation over the lifecycle of one year, which covers energy generation within this period.

#### 3.3.3. DES

$T_{c}$ is used to formulate levelized cost for the decentralized generation $D_{LCOE}$, which is calculated as the function of energy demand for decentralized energy and $T_{c}$ [3].

(18) $$D_{LCOE}=\mathrm{Load}\;\mathrm{demand}\times T_{c}.$$

### 3.4. Modeling the Types of Prosumers

In this section, prosumers are classified into two categories: critical prosumer (CP) and non-critical prosumer (NCP). CP is the prosumer that cannot be disconnected and has no role in the energy price negotiation, whereas NCP is the prosumer that operates when its energy offer is accepted as a demand or generation with a minimum price limit [51]. In Algorithm 1, the CPs must submit their estimated energy usage beforehand to the smart contract, while the smart contract calculates the energy gap. Energy gap is the difference between energy demand and energy generation, and it is denoted by energy losses or gains. If there are more energy demands, then energy generation offers will be accepted in a hierarchical order of the offered prices until there is excess energy generation. The acceptance of the energy offers is used to determine the energy prices for CP, whereas the NCPs are paid based on offered prices. The CPs pay the price for their energy usage and a reward is given to them if their estimated energy usage do not cause system imbalance. Similarly, a reward is given to NCPs if they can provide the estimated energy generation. Conversely, CPs get penalized if the estimated energy usage causes the system imbalance. The blockchain reward $R_{p}\left(h\right)$ during a given time slot *h*, where, subscript *p* denotes prosumer.

(19) $$R_{p}\left(h\right)=\frac{H_{p}\left(h\right)\times \left|E_{p}\left(h\right)-A_{p}\left(h\right)\right|\times \sum_{s=1}^{M}E_{p}\left(h\right)}{\sum_{s=1}^{M}E_{p}\left(h\right)+\sum_{s=1}^{M}A_{p}\left(h\right)},$$

(20) $$\begin{array}{c} H_{p}\left(h\right)=\left\{\begin{array}{cc} 1, & if\;WCR\left(h\right)>0\\ 0, & \mathrm{otherwise}, \end{array}\right. \end{array}$$

where $H_{p}\left(h\right)$ is the historical performance of prosumer during a given time slot *h* with work capital ratio (WCR) as the constraint, which is defined in Section 3.8. $E_{p}\left(h\right)$ is the prosumer’s estimated energy usage during a given time slot *h*, whereas $A_{p}\left(h\right)$ is the prosumer’s actual energy usage during a given time slot *h* and *M* is the cardinality of prosumers. The penalty $P_{p}\left(h\right)$ during a given time slot *h* of a given prosumer is defined as

(21) $$P_{p}\left(h\right)=\frac{|E_{p}\left(h\right)-A_{p}\left(h\right)|\times \sum_{s=1}^{M}R_{p}\left(h\right)}{|\sum_{s=1}^{M}E_{p}\left(h\right)-\sum_{s=1}^{M}A_{p}\left(h\right)|}.$$

**Algorithm 1:** Smart contract for SHS

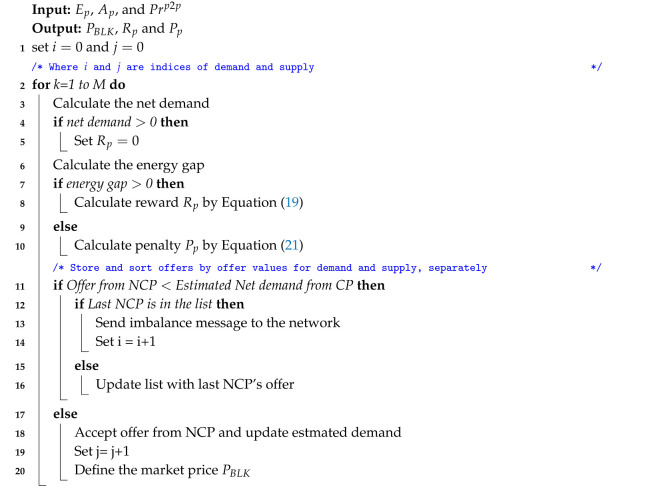



The blockchain price in Equation (22) during a given time slot *h* is calculated as the ratio of the accepted offered price $Pr^{p2p}\left(h\right)$ (where the superscript p2p denotes prosumer to prosumer), to the accepted energy ACTenergy during a given time slot *h*, and it is given as

(22) $$P_{BLK}\left(h\right)=\frac{\sum_{s=1}^{M}Pr^{p2p}\left(h\right)}{\sum_{s=1}^{M}ACTenergy\left(h\right)},$$

where $Pr^{p2p}\left(h\right)$ is the ratio of $E_{h}^{req}$ and $E_{h}^{val}$ given in Equation (23).

(23) $$Pr^{p2p}\left(h\right)=\frac{E_{p}^{req}\left(h\right)}{E_{p}^{val}\left(h\right)};\;E_{p}^{val}\left(h\right)\geq E_{p}^{req}\left(h\right).$$

### 3.5. Energy Trading of Prosumers in SSA

In this study, prosumers are considered in a given region as points that create a directed graph. Let $V_{p}$ be the vertices that denote prosumers and $E_{p}$ be the edges that connect prosumer *i* and *j*. The total transfer energy from prosumer *i* to *j* should not exceed their surplus energy $EXC_{p}^{p2p}$, which is defined as

(24) $$\sum_{i\in M}\sum_{j\in M}E_{p}^{T}\left(h\right)\times TE_{i,j}\left(h\right)\leq EXC_{p}^{p2p}\left(h\right);\mathrm{where}\;i\neq j,$$

where $TE_{i,j}$ is transmission energy decision of the prosumers, which is defined as

(25) $$\begin{array}{c} TE_{i,j}\left(h\right)=\left\{\begin{array}{cc} 1, & \;if\;\mathrm{prosumer}i\mathrm{can}\mathrm{send}\mathrm{energy}\mathrm{to}\mathrm{prosumer}j\\ 0, & \mathrm{otherwise}, \end{array}\right. \end{array}$$

where $E_{p}^{T}\left(h\right)$ is the energy that is transported from prosumer *i* to *j* during a given time slot *h*. To reduce energy loss during transportation, it is expected that the prosumers operate within their nearest locations. Finding the optimal path to move from one location to the other is not in the scope of this study.

In this study, a situation is considered where prosumers can instantly satisfy their actual energy needs with their on-site energy generations. As a result, a self-consumption $SC_{p}$ of a prosumer is examined in this study. $SC_{p}$ is the PV energy generation that is directly consumed by a prosumer, and the cost of individual PV energy generation is far less than the retail electricity, which makes $SC_{p}$ more profitable without subsidies [52]. Methods that can increase $SC_{p}$ of prosumers are energy storage and active load shifting as part of DSM [52]. $SC_{p}$ is defined as the ratio of PV energy generation that is directly consumed locally with battery storage over the total PV energy generation. In addition, $SC_{p}$ is formulated by taking the average of two $SC_{p}$ functions defined by [52,53], as $A_{1}$ and $A_{2}$, respectively.

(26) $$A_{1}=\frac{E_{dc}}{E_{dc}+E_{pv}},$$

(27) $$A_{2}=\frac{E_{dc}+E_{bc}}{E_{pv}}.$$

Thus, the proposed $SC_{p}$ is formulated as

(28) $$SC_{p}=\frac{A_{1}+A_{2}}{2};A_{1}\leq SC_{p}\leq A_{2},$$

where $E_{dc}$ is the PV energy generation that is directly consumed locally, and $E_{pv}$ is total PV energy generation and $E_{bc}$ is the battery charging [53]. Note that there is no universal nomenclature in the formulation of $SC_{p}$ [52]. The degree of self-consumption in which on-site PV energy generation is sufficient to meet prosumers’ energy needs is defined as self-sufficiency $SS_{p}$. $SS_{p}$ is formulated by taking the average of two $SS_{p}$ functions defined by [52,53], as $B_{1}$ and $B_{2}$, respectively:

(29) $$B_{1}=\frac{E_{pv}}{E_{l}+E_{pv}},$$

(30) $$B_{2}=\frac{E_{dc}+E_{bc}}{E_{l}},$$

(31) $$SS_{p}=\frac{B_{1}+B_{2}}{2};B_{1}\leq SS_{p}\leq B_{2},$$

where $E_{l}$ is the total energy demand. $SS_{p}$ can be expressed as the ratio of total consumed PV energy generation over the total energy demand. Note that the $SC_{p}$ is normalized by $E_{pv}$, whereas $SS_{p}$ is normalized by $E_{l}$. By increasing PV generation relative to the energy demand, will always decrease $SC_{p}$, while $SS_{p}$ either will remain unchanged or increased [52]. Therefore, the minimized instantaneous energy consumption $I_{p}\left(h\right)$ of the prosumers during a given time slot *h*, where *H* is the total time, defined as [52]

(32) $$I_{p}\left(h\right)=\min_{h\in H}\left\{E_{l},E_{pv}\right\}.$$

The energy deficit of each prosumer that is satisfied via the blockchain network during a given time slot *h* is defined as [52]

(33) $$E_{p}^{p2p}\left(h\right)=E_{l}\left(h\right)-I_{p}\left(h\right).$$

If $E_{p}^{p2p}\left(h\right)>0$, then prosumer can purchase the required energy from another prosumer via the blockchain. Otherwise, prosumer satisfies his actual energy need using his on-site energy generation. If excess energy $EXC_{p}^{p2p}$ is actualized after meeting the immediate energy demand need of prosumer, then it will be sold to other prosumers via the blockchain. $EXC_{p}^{p2c}$ is defined as [52]

(34) $$EXC_{p}^{p2p}=E_{pv}\left(h\right)-I_{p}\left(h\right).$$

If $EXC_{p}^{p2p}>0$, then it implies that there is an excess of energy generation; otherwise, there is no excess of energy generation. On the other hand, another situation is considered, where a group of prosumers can instantly satisfy their energy demand needs from a group of prosumers in another blockchain. The group instantaneous energy consumption needs $g_{p}$ during a given time slot *h* is minimized as

(35) $$g_{p}\left(h\right)=\min_{h\in H}\left\{\mathrm{Total}\;\mathrm{loads},\mathrm{Total}\;\mathrm{PV}\;\mathrm{energy}\;\mathrm{generations}\right\},$$

As prosumers can interact with each other through the blockchain, a large group of energy demand and excess energy generations need to be satisfied and aggregated. Equations (36) and (37) define the energy demand and excess energy, respectively.

(36) $$E_{g}^{g2g}\left(h\right)=\mathrm{Total}\;\mathrm{loads}\left(h\right)-g_{p}\left(h\right),$$

(37) $$EXC_{g}^{g2g}\left(h\right)=\mathrm{Total}\;\mathrm{PV}\;\mathrm{energy}\;\mathrm{generations}\left(h\right)-g_{p}\left(h\right),$$

where the superscript g2g denotes group to group. If $E_{g}^{g2g}\left(h\right)>0$, then the group of prosumers purchase the required energy from another group of prosumers via blockchain. This process is described as a regional blockchain-based energy trading. In Africa, four joint regional grids have been established to pool energy resources together, such as southern African power pool, which is the largest. In addition, others are western African power pool, eastern African power pool, and central African power pool [54]. The operating cost $OC_{p}\left(h\right)$ of each prosumer during a given time slot *h* is defined as

(38) $$OC_{p}\left(h\right)=\mathrm{Total}\;\mathrm{energy}\;\mathrm{needs}\left(h\right)\times S_{p}^{p2p}\left(h\right)+\mathrm{Total}\;\mathrm{excess}\;\mathrm{energy}\left(h\right)\times B_{p}^{p2p}\left(h\right),$$

where $S_{p}^{p2p}\left(h\right)$ is selling cost and $B_{p}^{p2p}\left(h\right)$ is the buying cost, which are calculated using Equations (39) and (40), respectively.

(39) $$S_{p}^{p2p}\left(h\right)=P_{BLK}\left(h\right)\times \frac{E_{p}^{p2p}\left(h\right)}{EXC_{p}^{p2p}\left(h\right)},$$

(40) $$B_{p}^{p2p}\left(h\right)=Pr^{p2p}\left(h\right)\times \frac{E_{p}^{p2p}\left(h\right)}{EXC_{p}^{p2p}\left(h\right)}.$$

Depending on the regional energy needs, groups of prosumers can act as regional energy sellers or buyers, which are described in Section 3.5.1 and Section 3.5.2.

#### 3.5.1. Regional Sellers of Energy

In this subsection, a case is considered where group of prosumers in a particular region tries to sell their excess energy to group of prosumers in other regions that have energy deficits through blockchain. The selling cost $S_{r}^{g2g}$ during a given time slot *h* for a particular region *r* is defined as

(41) $$S_{r}^{g2g}=\frac{\mathrm{Total}\;\mathrm{cost}\;\mathrm{of}\;\mathrm{energy}\;\mathrm{demand}\;\mathrm{within}\;\mathrm{the}\;\mathrm{region}\;\mathrm{at}\;h\;+\;\mathrm{The}\;\mathrm{total}\;\mathrm{cost}\;\mathrm{of}\;\mathrm{excess}\;\mathrm{energy}\;\mathrm{within}\;\mathrm{the}\;\mathrm{region}\;\mathrm{at}\;h}{\mathrm{Total}\;\mathrm{energy}\;\mathrm{generated}\;\mathrm{within}\;\mathrm{the}\;\mathrm{region}\;\mathrm{at}\;h},$$

where,

(42) $$\begin{array}{c} \mathrm{Total}\;\mathrm{cost}\;\mathrm{of}\;\mathrm{energy}\;\mathrm{demand}\;\mathrm{within}\;\mathrm{the}\;\mathrm{region}\;\mathrm{at}\;h\;=\\ \mathrm{Energy}\;\mathrm{demand}\;\mathrm{within}\;\mathrm{the}\;\mathrm{region}\;\mathrm{at}\;h\;\times \mathrm{Consensus}\;\mathrm{price}\;\mathrm{within}\;\mathrm{the}\;\mathrm{region}\;\mathrm{at}\;h, \end{array}$$

(43) $$\begin{array}{c} \mathrm{The}\;\mathrm{total}\;\mathrm{cost}\;\mathrm{of}\;\mathrm{excess}\;\mathrm{energy}\;\mathrm{within}\;\mathrm{the}\;\mathrm{region}\;\mathrm{at}\;h=\\ \mathrm{Excess}\;\mathrm{generated}\;\mathrm{energy}\;\mathrm{within}\;\mathrm{the}\;\mathrm{region}\;\mathrm{at}\;h\;\times \;\mathrm{Offer}\;\mathrm{prices}\;\mathrm{by}\;\mathrm{prosumers}\;\mathrm{within}\;\mathrm{the}\;\mathrm{region}\;\mathrm{at}\;h. \end{array}$$

#### 3.5.2. Regional Buyers of Energy

When a group of prosumers from blockchain in a particular region is unable to meet the energy needs of its region, it is expected by the group to purchase the needed energy from a group of prosumers from blockchain in other regions that have the excess of energy. The buying cost of regional blockchain $B_{r}^{g2g}$ is defined as

(44) $$B_{r}^{g2g}=\frac{\mathrm{Total}\;\mathrm{cost}\;\mathrm{of}\;\mathrm{energy}\;\mathrm{demand}\;\mathrm{within}\;\mathrm{region}\;\mathrm{at}\;h+\mathrm{Total}\;\mathrm{cost}\;\mathrm{of}\;\mathrm{excess}\;\mathrm{energy}\;\mathrm{within}\;\mathrm{region}\;\mathrm{at}\;h}{\mathrm{Total}\;\mathrm{load}\;\mathrm{at}\;h}.$$

### 3.6. Blockchain Incentive Mechanism

Several factors limit consumers’ willingness to pay for electricity and to set up the SHS project in developing countries. These factors include technical reliability like lack of information and the political influence, which is related to political disincentive. Therefore, a special incentive that motivates consumers to pay their electricity bills via blockchain is considered in this study.

**Figure 2:**
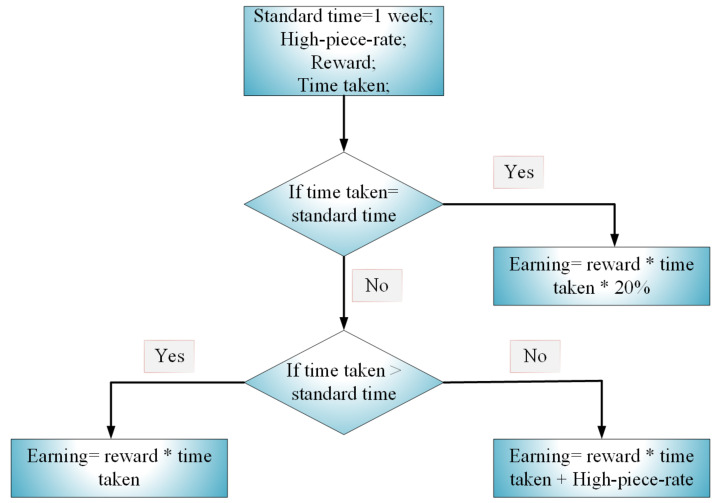
Flow diagram of smart contract for incentive mechanism for consumers.

In the proposed incentive scheme in Figure 2, a standard time frame is defined for the payment of electricity. The consumers’ historical performance is compared against the standard time to determine their efficiencies regarding their payment patterns. The Gantt task approach is used in this paper to incentivize consumers [55]. The Gantt task approach guarantees that the consumers’ time-rate (i.e., the amount of time it takes for consumers to make electricity payment) should be below the standard time. Thus, if a consumer takes longer time than the standard time to make the payment, then his efficiency is below 100%, and he is paid only the exact reward. Conversely, if a consumer completes payment at the exact standard time, then his efficiency is 100%, and in addition to his reward, a bonus of 20% will also be given to him. In contrast to the above, if a consumer completes payment before the standard time, then his efficiency is more than 100%; thus, in addition to his reward, he is paid at high-piece rate (i.e., an incentive given to consumers for each period of payment below the standard time).

### 3.7. Cryptocurrency Trading

In this paper, the rewards (coins) of prosumers are tradable between participants. Prosumers with excess coins can sell them to the blockchain and blockchain broadcasts these coins as cryptocurrencies to the network. Consumers who require additional earnings (incentives) can purchase these cryptocurrencies. Similarly, consumers can sell their excess earnings as cryptocurrencies with others who have low earnings. Depending on the policy, if NCP is unable to produce enough energy generation due to the cost of startup, then working capital (WC) offset will be used. This offset is the working capital (WC) cost reduction for each NCP. Furthermore, the same principle applies to the consumers: if a consumer does not have enough earnings to comply with the regulation of electricity bill payment, then electricity bill offset is used. Although, participants who take these benefits from this scheme will have to pay the exact offsets in the later time with their excess coins or incentives. Fraudulent participants will be blacklisted with a notification to all nodes within the blockchain network about their activities. Such participants can no longer get benefit from the scheme and their accounts will be deactivated.

The advantage of cryptocurrency trading is that there are clear WC cost and electricity bill reduction targets, which are to be achieved. Furthermore, the mechanism is also cost-efficient, as the cost of WC and electricity bill varies among the prosumers and consumers, respectively. This mechanism also makes it easy for participants to fulfill their financial obligations. In addition, this mechanism provides a way where excess coins or earnings can be efficiently utilized. With this mechanism, larger number of participants can be integrated to ensure a more stable market for energy trading and to encourage developing country’s involvement.

There are several important elements, which need to be considered when designing the proposed cryptocurrency trading.

1. Scope: it includes all the financial levels of prosumers and consumers and the type of RES.
2. Capital allocation: it determines the WC and the electricity bill reduction rate. It also determines the length of time that the cryptocurrency will stay relevant.
3. Offset policy: it determines whether the offset is necessary to be used in the scheme, what type of offset is allowed and the limit or amount of offset required.
4. Trading: it specifies the rules for cryptocurrency trading and the compliance policy.
5. Expansion potentials: it is flexible to accommodate larger number of participants and should be compatible with other monetary exchange.

### 3.8. WC Formulation

**Figure 3:**
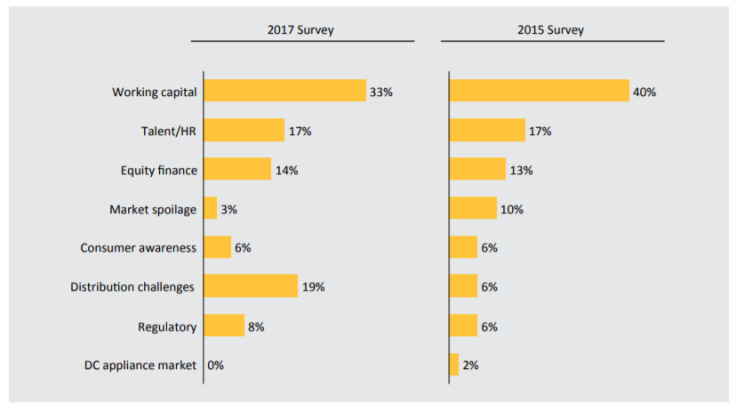
2017 survey: number of responses in each category when asked about top 3 barriers, n=19 (2017); 2015 survey: top 3 answers of polled respondents, n = 26 (2015) [56].

Globally, financial debts, technical, political, and managerial issues are the major causes of energy crises in developing countries. The total financial debt is expected to rise between $2.6 and $2.9 billion during the period of 2017 to 2022 [56]. However, new sources of finance are evolving, but they are not sufficient to fund larger needs. Thus, more funding is required to fulfill the WC of several investors. In addition, inadequate WC is forecasted to be the barrier to growth, as shown in a survey conducted in 2017 (see Figure 3). The survey report shows that WC has the highest percentage, which may restrict the growth of any organization. Therefore, the driving force behind the rise of valuations is enhanced by getting equity to finance WC. In addition, WC can be satisfied with external fundraising.

**Figure 4:**
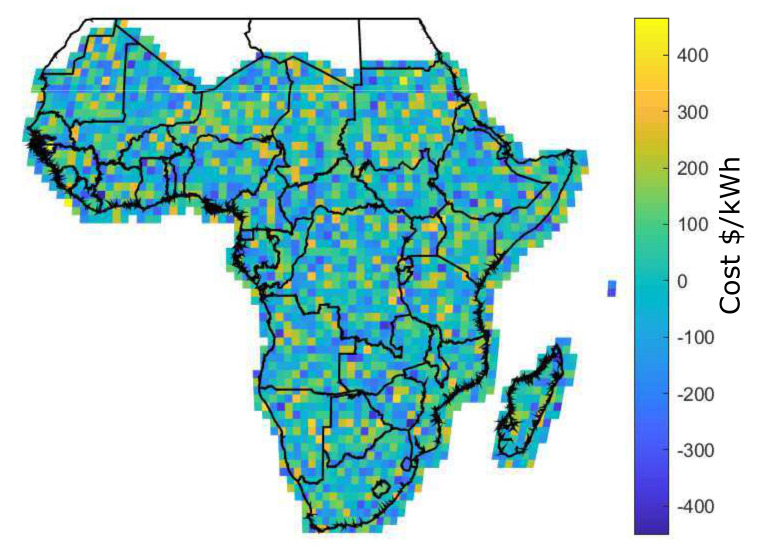
Working capital (WC) obtained from simulation.

WC management determines how the liquidity of business works and also ensures that proper planning and control of the current assets (CAs) and current liabilities (CLs) will relieve short-term responsibility risks [57]. WC management is used to measure the performance of a firm and should be made the constitutive part of the firm’s financial strategy. As shown in Figure 4, in east Africa, sales have greatly dropped across segment due to crosscutting issues, such as WC challenges, drought, and value-added task refund from the government. In Ethiopia, WC for suppliers and the available funds for the consumers are key challenges that restrict business growth [56]. More WCs are needed to offset the negative cash flow gap, especially when cash outflow is more than the cash inflow [58]. As shown in Figure 4, the exact financial records of prosumers for all locations in the SSA were not published; therefore, publicly available financial records in [56] (see Table 1) are used to generate random results for all locations. In Table 1, the first column presents the capital utilization, where receivable funding is the amount of money collected in a short time, inventories funding is an asset-based type of loan that allows you to leverage your inventory and capital expenditure is the amount of money spent on acquiring an asset. Their corresponding values in dollar ($) are given in the second column. The third column presents the capital source, such as operating cast, which measures the amount of cash generated from the normal business operation. Change in payable is the increase in receivables, while external debt is the amount borrow from foreign lender, external equity is where rate paid is equal to market rates and external grant is the non-payable fund given by foreign party. The corresponding values in ($) for capital source are presented in the fourth column.

The negative *WC* depicts that the *CLs* are more than the *CAs*. *WC*management is measured by the cash conversion cycle [57]. The *WC* can be calculated as

(45) WC=CA−CL,

where *CA* is the sum of receivable funding, inventories funding, and capital expenditure, whereas *CL* is the sum of operating cash, change in payables, external debt based on equity and grants. Thus, the *WCR* is given as

(46) $$WCR=\frac{CAs}{CLs};0<WCR\leq 1.$$

## 4. Simulations and Discussions

This section provides the discussion of simulation results.

### 4.1. Simulation Parameters

In this section, the assumption of economic parameters are presented in Table 2. This paper considers the same parameters and uses the linear regression model used in [19], to ensure a fair comparison. The international renewable energy agency describes the cost breakdown of SHS greater than 1 kW installed across Africa [19]. The solar insolation data used in this paper is obtained from the national aeronautic and space administration [59]. Table 2 gives the breakdown of the estimated economic assumptions considered in the scenario. In this table, the first column presents the names of the parameters, d.c denotes direct current, and soft costs are the interconnection costs; the second and third columns provide values for the current and future estimations. The proposed scheme is implemented using MATLAB, and blockchain is implemented using the Ethereum platform.

### 4.2. Evaluation of the Impact of Energy Reliability on Generation Cost based on FR

**Figure 5:**
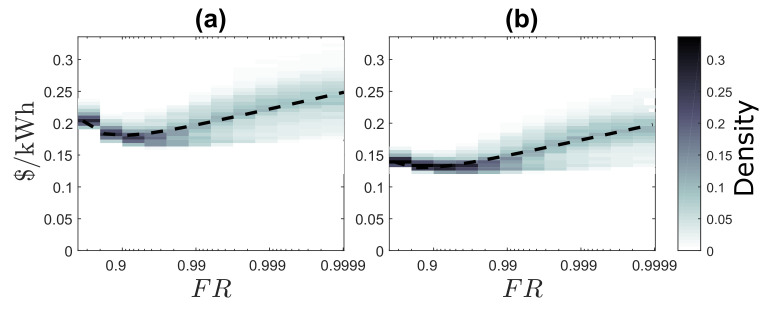
Relationship between FR and blockchain levelized cost of energy (BLCOE). (**a**) Levelized cost of energy (LCOE) without blockchain; (**b**) LCOE with blockchain.

Figure 5 shows the density of energy cost calculated within same locations for different values of FR for LCOE and the proposed BLCOE. Figure 5a shows the LCOE without blockchain [19], and Figure 5b shows the future scenario of BLCOE. The dashed line depicts the least-square fit to a single regression model for all locations, where a=−0.0257, b=0.0616, c=0.0839, and $R^{2}=0.4405$ for Figure 5a, and a=−0.0079, b=0.0189, c=0.0258, and $R^{2}=0.4305$ for Figure 5b. The scenario with LCOE shows a reduced reliability premium as well as reduced spatial variability in component costs. The reduced component costs invariably have non-uniform reductions in reliability premium for regions with high costs. On the other hand, the scenario with BLCOE shows that BLCOE rises logarithmically above 99% as FR approaches one. The minimum BLCOE confirms that the fixed cost associated with systems, such as inverters and wiring, have no significant impact as FR values approach one. Thus, it is economical to design the system with the blockchain concept, as blockchain does not alter the reliability even if FR is less than 90%. From the figure, the future scenario with blockchain shows the impact of the reduction in the reliability premium for the component costs with less spatial variability. If the reduction in the variance of BLCOE is less, then the component costs reduction may cause imbalanced impacts on the reliability premium for regions with high costs.

#### 4.2.1. Evaluation of the Energy Cost between Solar Modules and Battery Storage

**Figure 6:**
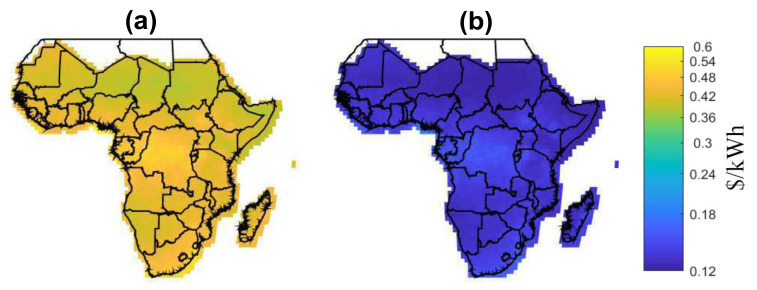
Comparison with different scenarios. (**a**) shows the current scenario without blockchain [19] and (**b**) shows the future scenario with blockchain.

Figure 6 shows the LCOE for two scenarios: Figure 6a shows the current scenario with LCOE [19] and Figure 6b shows the future scenario with BLCOE. Future scenario with BLCOE presents 95% decline in the cost of battery storage and 75% fall in the solar module and balance system costs. In addition, the anticipated future BLCOE varies across SSA by about 0.049 $/kWh with less spatial variability better than the present spatial variation of ~0.15 $/kWh [19]. The cost reductions in the future scenario minimize its coefficient of variation with uneven implications on the higher cost locations. The future scenario with blockchain confirms that the blockchain can provide further cost reductions via incentives and P2P energy trading.

#### 4.2.2. Comparison between of Isolation and Solar System Using Least-Cost System

**Figure 7:**
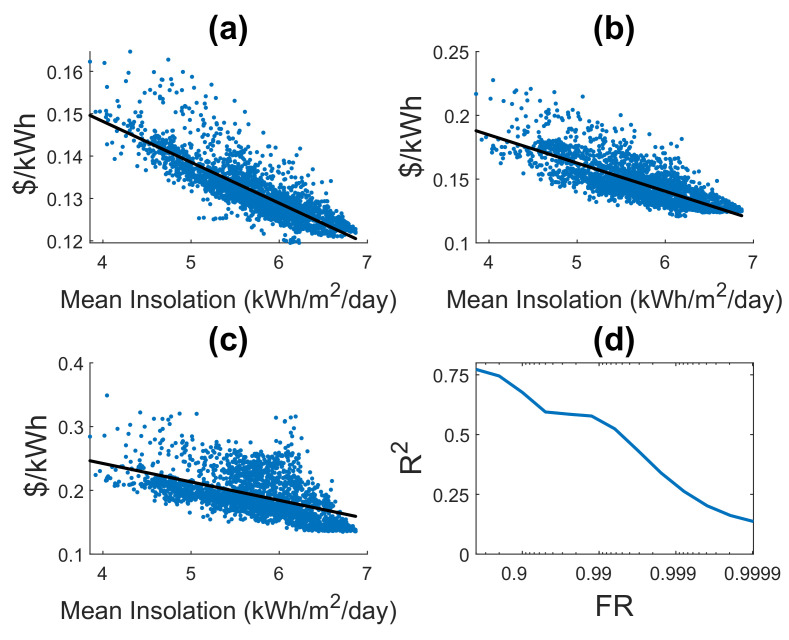
BLCOE with the predictive power of mean insolation. (**a**) shows the regression model onto mean isolation when FR=0.9 (**b**) shows the regression model onto mean isolation when FR=0.99 (**c**) shows the regression model onto mean isolation when FR=0.999 and (**d**) shows the regression model onto mean isolation for the different values of FR.

In Figure 7, results of the proposed BLCOE with a linear regression model denoted by a black line onto mean insolation are presented for different values of FR, for instance, Figure 7a when $FR=0.9,R^{2}=0.75$, it implies that the mean insolation achieves 75% of the variation of BLCOE for FR=0.9, Figure 7b when $FR=0.99,R^{2}=0.56$. It implies that the mean insolation achieves 56% of the variation of BLCOE for FR=0.99, and Figure 7c when $FR=0.999,R^{2}=0.23$, it implies that the mean insolation achieves 23% of the variation of BLCOE for FR=0.999. The results of Figure 7d shows that, as the values of FR increases, the regression values $R^{2}$ deteriorates accordingly [19]. The isoreliability optimization model achieves a better prediction of BLCOE based on mean insolation with a lower FR value. The converse is not true for higher values of FR, as more costs are obtained from the temporal variability of the solar resources than mean insolation. As shown in Figure 7a–c, the mean insolation loses its predictive power as FR values increase, contrarily to the proposed model in Figure 7d. The loss of prediction occurs because the variation of solar energy is reliable at high value of FR, rather than the mean insolation.

#### 4.2.3. Evaluation of Energy Reliability Premium in SSA

**Figure 8:**
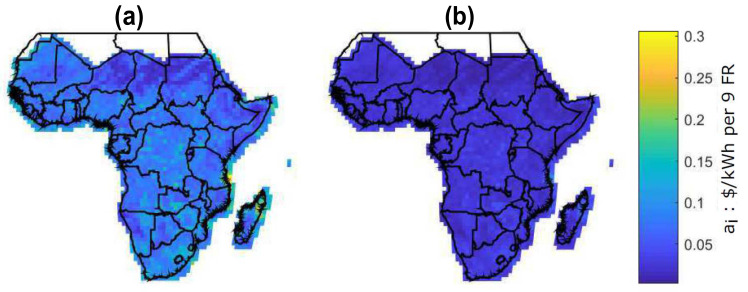
Reliability premium on the spatial distribution. (**a**) is the current reliability premium (**b**) is the future scenario with blockchain. Reliability premium $a_{i}$ is the coefficient in least-square fit to a model for all locations.

Figure 8 shows the reliability premium for all locations across SSA. In the current reliability premium (a), some portions of locations have a higher premium with cost above 0.05 $/kWh [19]. Conversely, the future scenario with blockchain shows uniform premium across the distribution with cost less than 0.05 $/kWh. The premium is associated with additional costs of storage and solar capacity. However, in the future scenario case, superiority in terms of costs of battery storage and solar module among locations is not observed.

#### 4.2.4. Variation of Load Demand with Respect to FR

**Figure 9:**
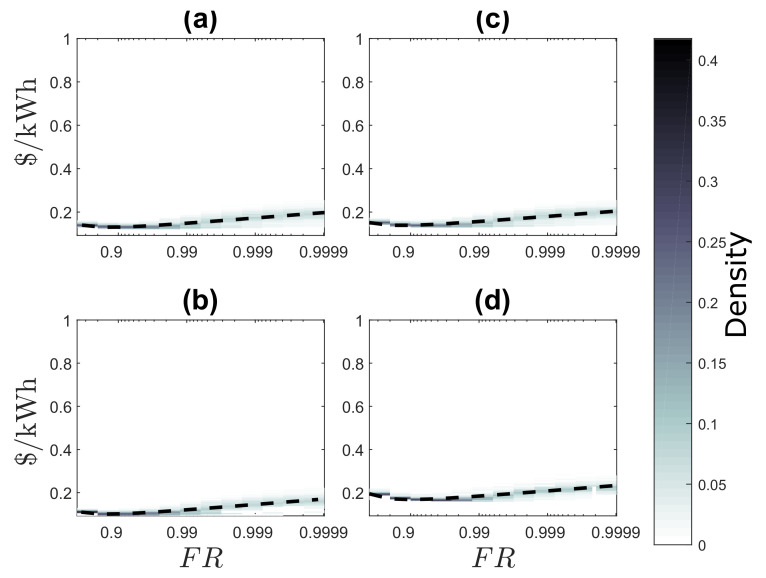
Comparison with different load demands. (**a**) shows the constant load demand (**b**) shows the heaviest day load demand (**c**) shows medium household load demand and (**d**) shows the heavy night load demand.

The effect of blockchain technology is analyzed based on the relationship between FR and different load demands as shown in Figure 9a–d. Figure 9a depicts the constant demand, Figure 9b is the heaviest day demand, Figure 9c is medium household demand, and Figure 9d is the heavy night demand. The figures report low BLCOE for various load demands. It is observed that 0.1 $/kWh cost difference for the heaviest day and heavy night demand is achieved, which depicts the boundary for achieving load shifting from day to night using the demand response. It is also observed that the medium demand roughly achieves the minimum cost as compared to constant demand. It is because the medium demand approximately balances consumption from the battery storage. Due to the decentralized and localized P2P energy trading activities between prosumers, the load shifting from high peak to low peak time slots using the demand response program is fully implemented. Blockchain provides benefits, such as real-time energy trading, fast response, and efficient payment vetting process with minimum or no delay.

#### 4.2.5. Prediction of the Future Costs with Respect to Isoreliability

**Figure 10:**
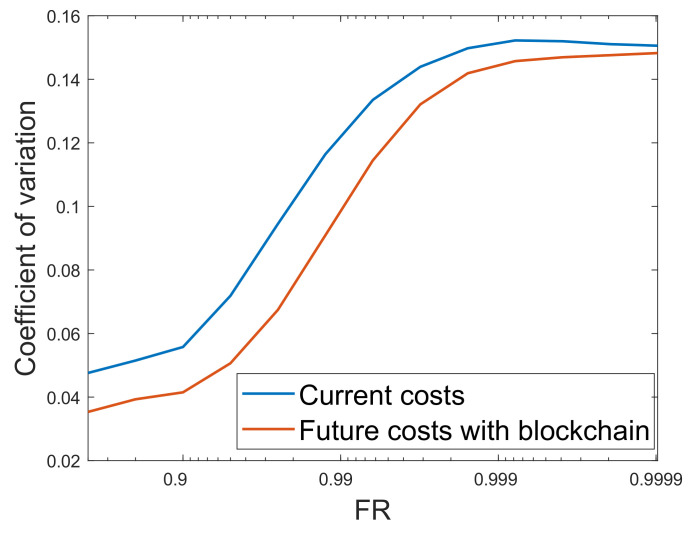
Current costs versus future costs with blockchain.

Figure 10 shows the effect of cost coefficient of variance on BLCOE. The coefficient of variation gives the ratio of the standard deviation to the mean, which is used for comparing two distributions whose measurements are incomparable. If the value of the coefficient of variation is high, it means that there is a high level of dispersion around the mean. However, a smaller value of the coefficient of variation denotes better estimate. From the figure, as the values of FR approach one, the coefficient of variation in BLCOE increases accordingly and reliability is achieved at the expense of generation cost. In addition, the future cost of blockchain based scenario outperform the current cost in terms of minimum coefficient of variation. The lower values of the coefficient of variation of the proposed scenario shows the accurate estimate for the future energy cost.

#### 4.2.6. Comparison of the Different Cost Systems

**Figure 11:**
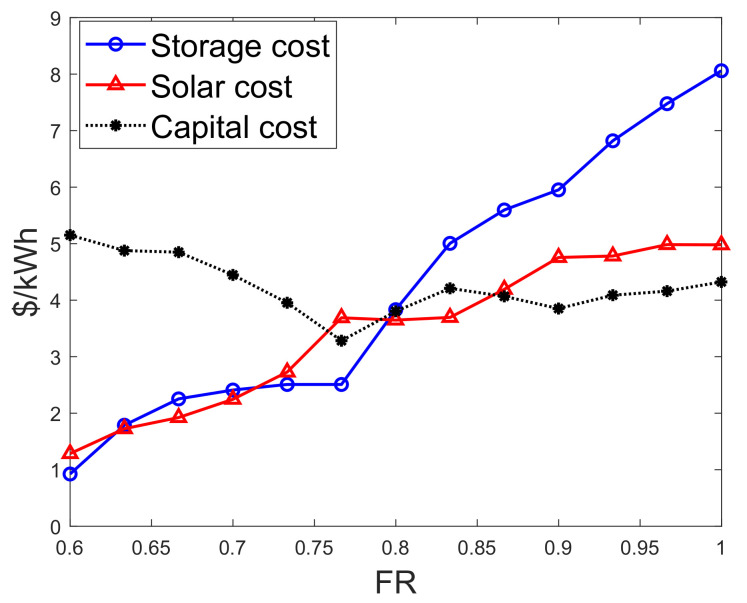
Energy costs versus FR.

Figure 11 shows the cumulative cost of solar generation and battery storage versus FR. From the results, it is observed that as FR increases from 60 to 77%, capital cost reduces with the cost of storage and solar generation using Equations (10), (11), and (15). It implies that with a smaller population, storage and solar generation can satisfy prosumers’ load demands within a blockchain-based decentralized infrastructure. However, as FR increases from 77 to 100%, costs of solar generation and battery storage increase rapidly, whereas capital cost slowly rises and then falls.

#### 4.2.7. Least-Cost Evaluation for CES, DES, and BDES Contributions to Electrification in SSA

**Figure 12:**
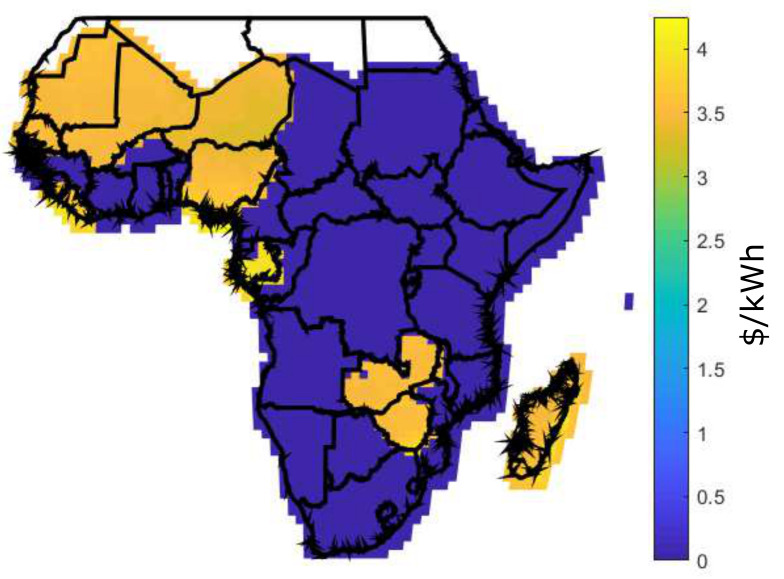
Decentralized energy system (DES).

**Figure 13:**
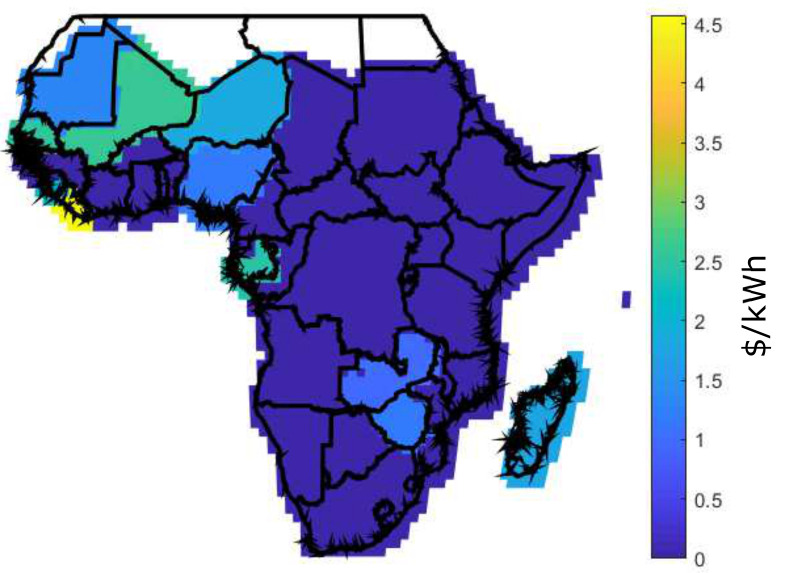
Centralized energy system (CES).

**Figure 14:**
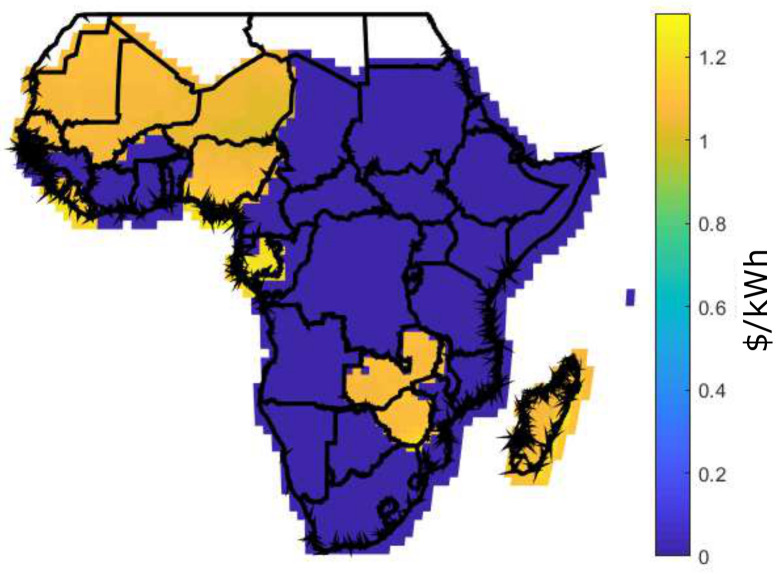
Blockchain-based decentralized energy system (BDES).

In Africa, access to affordable energy from the grid requires huge cost and long-term development. As a result, affected regions depend on a solar energy project that remains unfunded; however, blockchain-based project initiative can bridge the gap. Due to the high cost of PV cells, solar panels are built only when a sufficient amount of solar cells are purchase. Considering the households without solar grid, the actual owner of solar cells receives cryptocurrency as payment for the energy sold. In this study, analysis of rural electricity generation option in SSA has assumed a daily energy demand to be 8.2 kWh. Also, the energy generation of 100 kWh per month is shown in Figure 12, Figure 13 and Figure 14 using Equations (16), (17), and (14), for DES, CES, and BDES, respectively.

It is observed from these figures that some regions like Morocco, Algeria, Libya, Nigeria, Gabon, Zambia, and Zimbabwe have high energy generation cost, which is above 2.5 $/kWh for the decentralized infrastructure as compared to CES and BDES. However, BDES outperforms its counterpart with the minimum energy generations cost. The high energy generation cost for DES occurs as a result of retail electricity price, prosumers’ incomes, and quantity of energy demand. For a fair comparison, same FR value is used for DES, CES, and BDES. However, there are no retail electricity prices at 100 kWh per month for some countries, as they are not being published.

The retail electricity prices of 100 kWh per month are shown in Table 3 for FR=60% and Table 4 for FR=99%, respectively. The first columns from both tables present the lists of selected 43 countries in Africa. The second set of columns present the retail prices at 100 kWh per month; the third set of columns present the fraction of the population in percentage; and the fourth, fifth, and sixth sets of columns present the results for CES, DES, and BDES, respectively. In addition, transmission levelized cost parameters are taken from [3]. From Table 3, it is observed that more than 80% cost of energy generations for centralized infrastructure are low as compared to decentralized infrastructure, and the blockchain-based decentralized infrastructure outperforms its counterpart due to its lowest energy generation cost. It is because the estimated cost of energy consumption varies significantly from country to country. The comparative analysis of these infrastructures is based on mean and standard deviation (Std) of energy generation. Lowest mean values of the energy generation cost indicates that the infrastructure is far better than infrastructures with the highest mean values. From the results in Table 3, when FR=60%, it is clear that countries like Burkina Faso, Cote d’Ivoire, Gambia, Guinea, Liberia, Mali, and Senegal support DES with low mean values of the generation cost as compared to the CES.

Burkina Faso is a country that has long recognized decentralization by enacting an act in 1998 for rural development. In terms of electricity, Burkina Faso relies much on thermal-fossil generation, which generated ~70% of the country’s total electricity. With regards to RES, Burkina Faso solar grid is based on SHS and hybrid PV-diesel mini-microgrid, which has installed capacity of ~10 kWh in 2014 [60]. This generation formed ~0.1% of the country’s total energy consumption. In 2017, solar grid capacity of 33 MW was installed, which contributed to ~5% of the country’s total electricity production. Solar grid with a capacity of 100 MW was installed in 2018, which generated about 10% of the total country’s electricity [60]. The simulation results confirm that Burkina Faso can achieve the mean generation cost of 0.9129 $/kWh for CES, 0.8218 $/kWh for DES, and 0.4205 $/kWh with BDES.

Gambia is a country with a total electricity generation of 100 MW and excess demand of about 50 MW. In Gambia, ~42% of Gambians have access to electricity, whereas 58% of the population of Gambians have no access to electricity [61]. Fossil fuels produce about 98% of the electricity production of the Gambia, while RES with a series of potential options is underexplored. Solar system is the most considered option, which has a production capacity of about 4 kWh/m^2^ of solar radiation [61]. Although, there is hope to increase the solar generation from 2 to 4% by the national development plan [61]. However, the simulation results confirm that the Gambia can achieve the mean generation cost of about 2.4512 $/kWh for CES, 2.2143 $/kWh for DES, while 1.1332 $/kWh per month for BDES.Guinea-Bissau is a country with an energy mix of fossil fuels, biomass, and hydropower. The primary source of electricity is the biomass; however, majority of the population have no access to electricity [62]. From 2012 to 2014, ~1% access to electricity was recorded in Guinea-Bissau, which resulted from poor energy performance and social instability [62]. With regards to RES, there is no statistical information as the government of Guinea-Bissau regards RES as a discrete sector [62]. The simulation results confirm that Guinea-Bissau can achieve the mean generation cost of 0.9486 $/kWh for CES, 0.7975 $/kWh for DES as compared to 0.4081 $/kWh per month for the BDES.

Cote d’Ivoire is a country with electricity accessible by 80% of urban area and about 29% of rural community. Approximately 56% of the nation’s electricity rate is generated from hydropower, natural gas, and biomass. With regards to RES, a mini-grid based on solar generation with a capacity of about 2077 kWh/m^2^ was installed [63]. Note that Cote d’Ivoire has no current operational solar grid; however, a standalone solar system is used for domestic lighting [63]. The simulation results confirm that Cote d’Ivoire can achieve the mean generation cost of 0.6105 $/kWh for CES and 0.3595 $/kWh for DES, whereas 0.1840 $/kWh per month was achieved for the BDES infrastructure.

In Liberia, biomass is used as the sole source of energy consumption, which creates a share of 80% of the used energy. Approximately 16.8% of urban dwellers and 2% of rural settlers can access electricity from self-energy generation with diesel and gasoline generators [64]. With regards to RES, monthly solar radiation generates ~4 kWh/m^2^/day of electricity during the raining seasons, whereas 6 kWh/m^2^/day is generated during the dry seasons [64]. The energy generations from solar can achieve the average total capacity of 1712 kWh/m^2^ of electricity. The simulation results confirm that Liberia can achieve the mean energy generation cost of approximately 4.0300 $/kWh for CES and 2.4216 $/kWh for DES as compared to 1.2393 $/kWh per month for BDES.

In Mali, electricity generation is dominated by 55% of hydraulic and 45% of diesel energy sources. Solar generation capacities are recorded with ~2100–2300 kWh/m^2^, which increase the country’s total energy generation [65]. In 2010, 6.3 MW of energy was generated from RES, whereas an estimated value of approximately 150.7 MW for 2020 and 201.8 MW for 2030 is expected [65]. The simulation results confirm that Mali can achieve a mean generation cost of 2.2928 $/kWh for CES, 2.1995 $/kWh for DES as compared to 1.1256 $/kWh per month for BDES.

Senegal electricity is dominated by 87.4% of fossil fuels and 3.3% of RES [66]. However, from the simulation results, it is confirmed that Senegal can achieve mean generation cost of 2.4818 $/kWh for CES, 2.1963 $/kWh for the DES as compared to 1.1240 $/kWh per month for the BDES.

It is also observed that the proposed blockchain-based infrastructure shows a minimum mean energy generation cost for a retail price of 100 kWh per month as compared to CES and DES, respectively. The performance of the BDES is shown in Figure 14. The proposed incentive mechanism further reduces cost of energy generations. From the simulation results in Table 4, it is also observed that when FR=99%, decentralized infrastructure no longer provide energy generation cost reduction. With the BDES, countries can achieve relatively minimum mean energy generation cost.

### 4.3. Evaluation of Blockchain-Based Energy Trading in SSA

**Figure 15:**
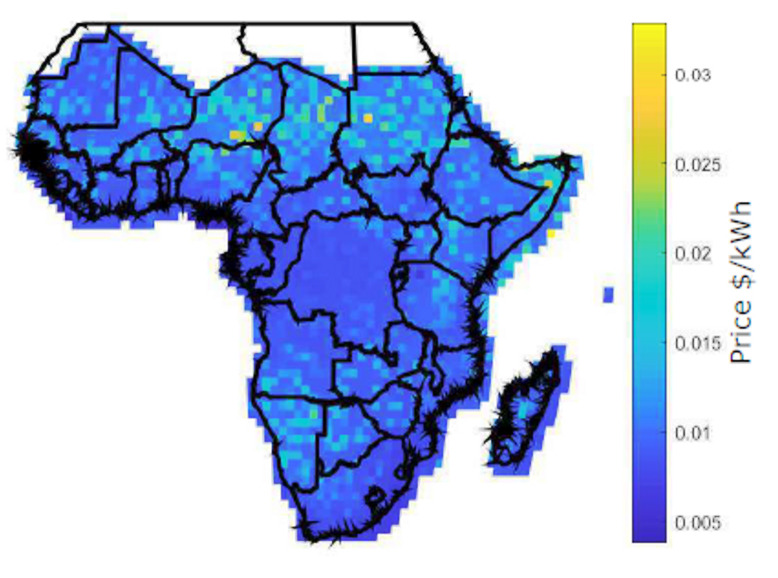
Blockchain price at 250 kWh per month.

Figure 15 shows the spatial distribution of the proposed blockchain prices across all locations for 2400 prosumers using Equation (22). The result shows that only a small portion of locations has a high price, which is due to variations in the accepted offer and accepted energy.

**Figure 16:**
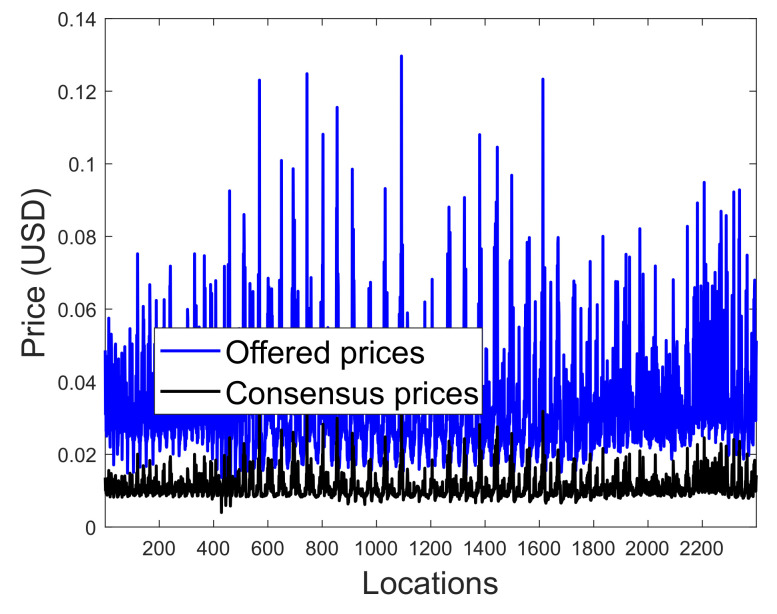
Offered price versus consensus price.

Figure 16 shows the result of the offered prices versus consensus prices across all locations. The offered prices are given by the NCPs while the consensus prices are obtained using Equation (22). The figure shows that the consensus prices are lower than the offered prices and it is due to the negotiation by all NCPs.

#### 4.3.1. Evaluation of Different Unmet Loads for Blockchain-Based Energy Trading

To examine the performance of the proposed blockchain-based energy trading mechanism. Four different unmet load consumption cases, such as constant, medium hourly, heavy night, and daily home business are considered for the analysis.

**Figure 17:**
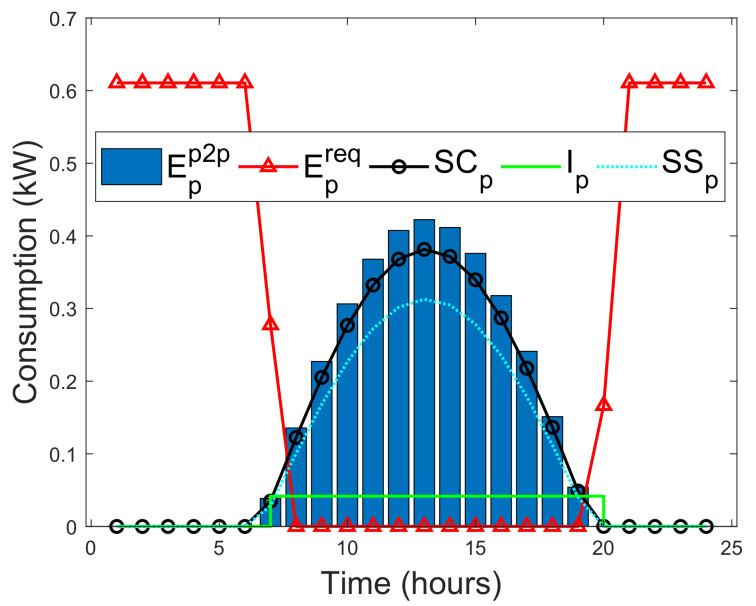
Constant hourly household consumption.

Figure 17 shows the constant hourly load consumption using Equations (28) and (31). It is observed that during the time slots of 1 am–6 am, the maximum unmet energy load is 0.6 kW. However, between the time slots of 8 am–19 Pm, there is no energy demand, which implies that the load demands are satisfied by self-consumption. During these time slots, the battery will be in charging state. Note that DSM can be applied to satisfy the unmet load through load shifting and self-consumption, while the excess of the energy after self-consumption will be sold to other prosumers via blockchain with an offered price of 6.60 $/kWh. It is also observed that as the prosumers’ unmet loads increase relative to the energy generation, self-sufficiency of the prosumers decreases with increase in self-consumption. Therefore, using Equation (32), the instantaneous consumption of the prosumers either decreases or remains constant.

**Figure 18:**
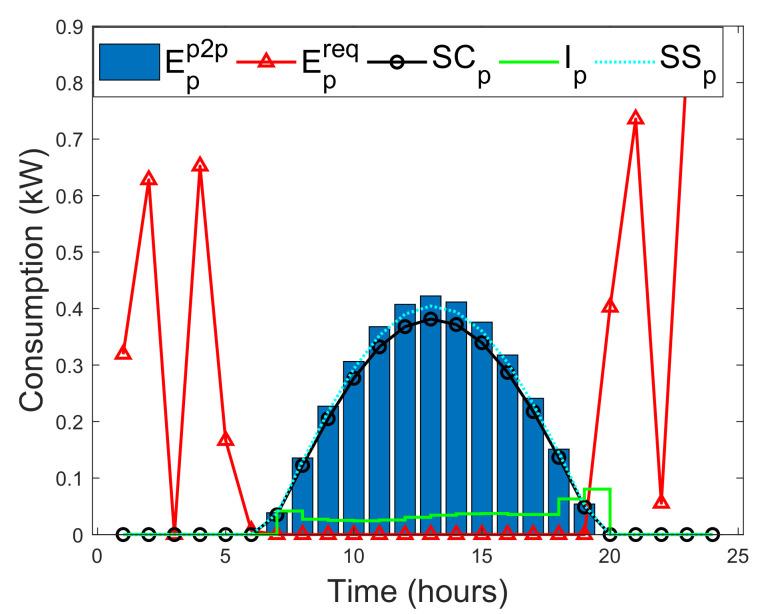
Medium hourly household consumption.

Figure 18 shows the unmet load for medium household load consumption using Equations (28) and (31). It is observed that during the off-peak time slots, maximum unmet load is 0.85 kW. During the peak time slots, prosumers load demands are satisfied by self-sufficiency. It is also observed that by increasing the energy generation relative to unmet loads, will reduce the self-consumption, while self-sufficiency will be increased. The excess of energy after self-consumption will be sold to the blockchain at an offered price of 7.58 $/kWh.

**Figure 19:**
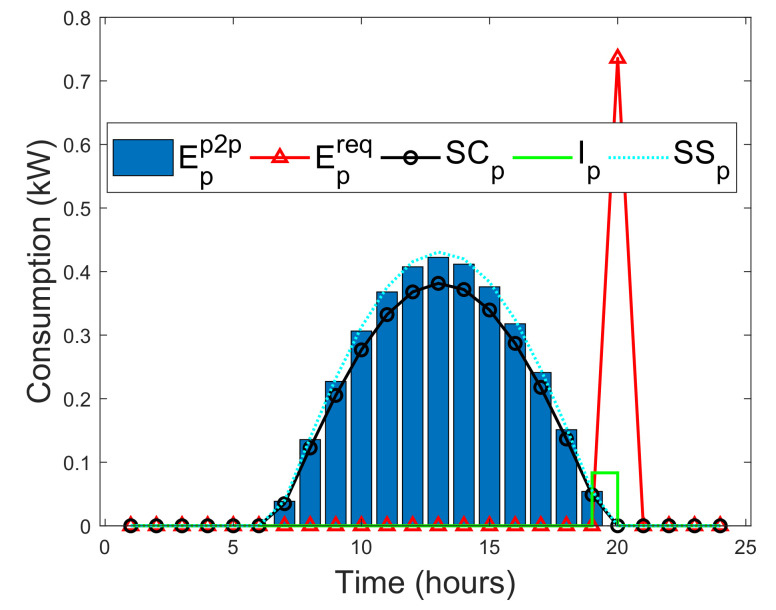
Heavy night hourly household consumption.

Figure 19 shows the unmet load for heavy night hourly household consumption using Equations (28) and (31). It is observed that at time slot 20:00, the maximum unmet load is 0.75 kW. The excess of energy after self-sufficiency will be sold to the blockchain with an offered of 9.84 $/kWh.

**Figure 20:**
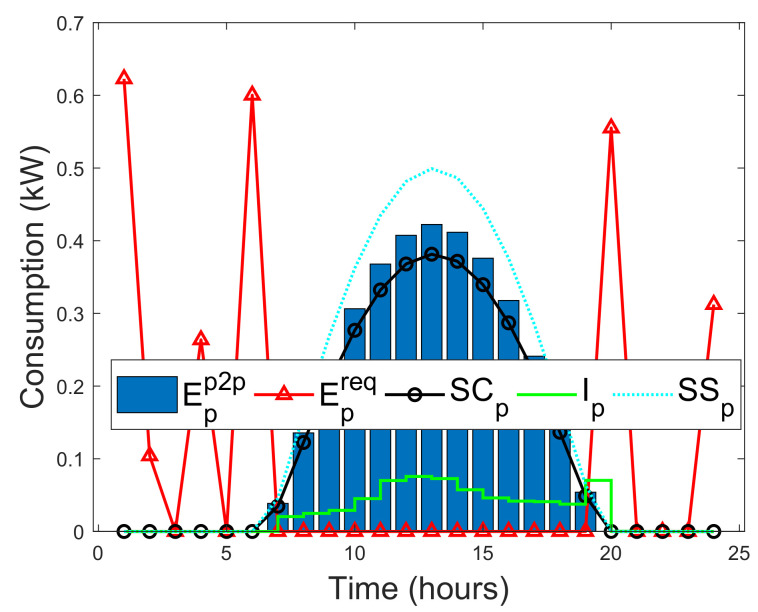
Home business daily household consumption.

Figure 20 shows the unmet load for daily business household load consumption using Equations (28) and (31). It is observed that the maximum unmet load is 0.65 kW. The large margin between self-sufficiency and self-consumption implies that it requires extra energy generations to satisfy the unmet loads. Note that the offered price for the energy sold to the prosumer via blockchain is 23.63 $/kWh.

#### 4.3.2. Comparison between Selling Cost and Buying Cost for Blockchain-Based Energy Trading

Table 5 shows the summary of annual hourly and daily trading results of the whole prosumers network with different unmet load consumption profiles using Equations (39) and (40). The first column in the table gives the variety of unmet loads; the second, third, and fourth columns present the selling cost, buying cost, and the offer price, respectively; and the fifth column presents the proposed blockchain price. From the results, the selling cost of prosumer (i.e., prosumer with excess of energy generation) is found to be far less than the buying cost of the prosumer (i.e., prosumer with energy deficit). It is because each prosumer, determines the offered price based on the cost of on-site energy generation using Equation (23). On the other hand, based on consensus mechanism, a minimum negotiation price is reached by all mining nodes in the blockchain network. It also determines the selling price for the amount of energy required by the prosumers. However, the minimum blockchain price may raise the cost of storage for buying prosumers as they take this advantage to buy more energy. Note that this problem can be solved using a proportional sharing approach. This approach will limit the amount of energy required by each prosumer per day, which depends on the policy stipulated in the smart contract. However, this study is not limited to proportional sharing approach, other fair allocation mechanisms, such as game theory can also be applied.

## 5. Research Implications

As blockchain technology is underexplored in SSA, this study provides important research implications in four aspects: (1) The benefit of blockchain technology to address lack of trust, security and privacy in SSA. (2) The use of blockchain-based energy trading mechanism in SSA. (3) The use of blockchain-based cost-effective energy planning model for decentralized electrification in SSA. (4) The benefit of the proposed blockchain-based incentive mechanism for customers in SSA.

First, until recently, most energy transactions in SSA have been conducted in a centralized manner, which has legal and technical implications. In this framework, distributed transactions are achieved effectively in real-time. The proposed framework offers trust consolidation for distributed transactions, as all nodes are in agreement to verify and authenticate transactions before they are encapsulated into the blockchain. With the proposed framework, historical antipathy is eliminated using the blockchain’s smart contract. Critical energy infrastructures in SSA are weak and unreliable in terms of energy security and management as compared to developed countries. Therefore, the proposed BDES is reliable and can strengthen energy infrastructures by its underlying security properties of the blockchain technology. Second, energy generations in SSA are insufficient to satisfy the increasing energy demands of the region, especially in peak time demands. The proposed blockchain-based energy trading mechanism allows prosumers with surplus energy to sell their excess energy to other consumers with insufficient energy at a low selling price. Figure 17, Figure 18, Figure 19 and Figure 20 show how Equations (28) and (31) are used efficiently by prosumers to instantly satisfy their actual energy demands with their on-site energy generations. Excess of energy after self-consumption is traded with other prosumers with insufficient energy. Different unmet load demands are considered in this study that indicate the resilience of the proposed mechanism to handle variety of unmet load demands. Table 5 shows that prosumers can use Equations (39) and (40) to calculate the energy selling and buying costs. Computation of both costs depends on prosumers’ energy demand and excess energy. The offer prices by prosumers are obtained from the price negotiation mechanism of Equation (23). Third, lack of cost-effective energy planning model in SSA has resulted in high cost of OM in transmission and distribution. Therefore, the proposed BLCOE is used to measure the impact of energy reliability on generation cost based on FR. Results of Figure 5, Figure 6, Figure 7 and Figure 8 show that prosumers can achieve low energy costs, if the proposed mechanism is implemented. Additionally, low reliability premium is achieved with minimum BLCOE for prosumers with less variability in the component cost using Equation (15). Furthermore, using Equations (14), (16), and (17), countries can decide which models best suit their energy needs. However, the proposed solution of Equation (14) outperforms Equations (17) and (16) with the least energy generation cost. Table 3 and Table 4 confirm that the proposed BDES achieves minimum energy generation cost for 60% and 99% of the population in SSA with retail price of 100 kWh per month. Fourth, energy theft in SSA increases the financial burden of the energy prosumers as customers refuse to pay for the electricity they have consumed. Therefore, the proposed incentive and cryptocurrency trading mechanisms will manage excess rewards of customers and offset their financial debts.

Future research could examines how socio-cultural background of prosumers in SSA may limit the acceptance of the proposed framework as the adoption of blockchain technology is still in its infancy. Additionally, the economic background of prosumers in SSA is another constraint that can prevent the proposed system from being implemented, as prosumers need to be trained and well informed about the emerging technology.

## 6. Conclusions

The proposed framework in the paper efficiently resolves the possible security challenges of SG for the decentralized electrification in SSA, such as lack of trust and privacy of prosumers’ data. The solutions provided by the proposed framework are threefold. First, as cost-effective energy planning model in SSA is underexplored, this paper proposes a BLCOE mechanism that compares the energy cost of solar module and battery storage for standalone SHS in SSA. In the proposed mechanism, the impact of energy reliability on generation cost is measured based on FR and SL. BLCOE is minimized by approximately 95% for battery and 75% for the solar module. Future energy costs vary across SSA on an average of 0.049 $/kWh FR against existing cost model of 0.11 $/kWh. The impact of reliability premium is as low as 0.05 $/kWh in the anticipated blockchain scenario using the least-cost system. The low impact of reliability premium observed from the simulation results enables prosumers to make upfront curtailment decisions in real-time. The impacts of BLCOE on the variation of consumers’ load demands, such as constant demand, heaviest demand, medium demand, and heavy night demands, are also studied. The simulation results confirm that a minimum BLCOE is achieved for different loads, which occur based on demand response. However, the heavy night demand incurs a high BLCOE cost. The coefficient of variation is used to compare the distributions of the proposed cost model with the existing model. Results from the coefficient of variation of the proposed model confirm the accurate estimate for future energy cost.

Second, because of weakness of the existing energy infrastructures in SSA to generate reliable energy that can satisfy the growing energy demands in the region, this paper proposes a method that pools RES together from different prosumers using the proposed self-consumption and self-sufficiency methods. To ensure that the excess energy of prosumers is not wasted, a price negotiation mechanism for prosumers is proposed. In the proposed mechanism, two types of prosumers are considered: NCPs and CPs. NCPs engage in price negotiation by contributing their excess energy to the blockchain-based energy market, whereas CPs have no role in the price negotiations, but can request for energy to satisfy their current demands. A reward is given to NCPs based on their energy contributions, while penalties are issued to CPs if their requests cause the system imbalance. Simulation results show that the minimum consensus prices are achieved with price negotiation. In addition, regional blockchain-based energy trading is achieved.

Third, electricity theft is predominant in SSA, as customers may refuse to pay for electricity they have consumed. These attitudes of customers increase the financial burden on the utility company. Therefore, this paper proposes an incentive mechanism based on Gannt task approach to motivate customers to pay their electricity bills. Additionally, excess rewards from customers can either be traded within the blockchain network or used for settlement of customers’ financial debt.

This study will enable agencies and governments to make a strategic decision about, where and when they should rely on the BDES to meet societal goals. The proposed mechanism will also be useful to the independent power producers to address the power shortage problem by offsetting their WC cost through the sale of cryptocurrency. The proposed framework serves as a guide to set up country-to-country energy trading through the blockchain technology. In addition, this study aims to address the following research questions.

- Is there any feasible long-term cost-effective energy planning model for energy generation in SSA? The framework proposed in this paper provides energy cost planning model that quantifies the impact of reliability on energy cost based on FR. Also, the proposed model estimates the future energy generation cost for 20 years to be 0.049 $/kWh as compared to 0.11 $/kWh for the existing scheme.
- Are there any solutions to historical antipathy or lack of trust and security that can jeopardize the process of regional decentralized energy cooperation in SSA? The proposed framework ensures that a trustful and secure environment is established for cooperation among regional decentralized energy providers. Also, with blockchain smart contract, historical antipathy among regions is eliminated.
- Are there any technologies that provide a trade arrangement for cross-border electricity and can effectively solve the problem of fair energy distribution and payment of electricity bill in SSA? The proposed framework ensures that blockchain-based cross-border energy trading is achieved. Also, the use of cryptocurrency as means of payment is encouraged, which minimizes the vetting process and eliminates the third party system.

In future, this study intends to analyze the possible security threats that may confront the proposed framework and offer the required remedies. In addition, this study intends to examine the impacts of the increasing number of prosumers and consumers on the performance of the proposed framework, for example throughput, computational cost and storage.

## Figures and Tables

**Table 1:** Financial needs of off-grid solar affiliated to companies, which is projected from 2017 until 2022 [56].

Capital Utilization	Values ($)	Capital Source	Values ($)
Receivable funding	3350–3725	Operating cash	900–1000
Inventories funding	1275–1425	Change in payable	225–250
Capital expenditure	475–550	External debt	2600–2850
		External equity	1175–1325
		External grants	200–275

**Table 2:** Assumption of economic parameters [19].

	2017	Future (2022)
**Costs of solar**		
Module + d.c balance of system	1.00	0.50
Charge controller	0.20	0.10
Total ($/W)	1.20	0.60
**Costs of battery**		
Total ($/kW)	400	100
**Costs of load**		
Inverter	0.30	0.15
Soft costs + d.c balance of system	1.00	0.50
Total ($/W)	1.30	0.65
**Other assumptions**		
Operation and Maintenance	100 $/kW	
Length of project	20 years	
Battery replacement	10 years	
Discount	10%	

**Table 3:** Comparison of the different energy generation costs when FR=60%.

Country	Retail Price at 100 kWh/month	*FR*	CES ($/kWh)		DES ($/kWh)		BDES ($/kWh)	
			Mean	Std	Mean	Std	Mean	Std
Angola	0.04	0.6000	0.0681	0.2424	0.1629	0.5805	0.0834	0.2971
Benin	0.30	0.6000	0.4519	0.6431	0.7998	1.0977	0.4093	0.5617
Botswana	0.10	0.6000	0.1445	0.3678	0.3056	0.7761	0.1564	0.3972
Burkina Faso	0.31	0.6000	0.9129	1.2008	0.8218	1.0667	0.4205	0.5459
Burundi	0.00	0.6000	0.0000	0.0000	0.0000	0.0000	0.0000	0.0000
Cameroon	0.10	0.6000	0.2921	0.5823	0.5239	0.9731	0.2681	0.4980
Central African Republic	0.00	0.6000	0.0000	0.0000	0.0000	0.0000	0.0000	0.0000
Chad	0.18	0.6000	0.1665	0.5073	0.2102	0.6362	0.1075	0.3256
Congo	0.00	0.6000	0.6634	1.0723	0.6988	1.1298	0.3576	0.5782
Congo drc	0.12	0.6000	0.0862	0.2702	0.2071	0.6488	0.1060	0.3320
Co´te d’ivoire	0.27	0.6000	0.6105	1.4788	0.3595	0.8475	0.1840	0.4337
Djibouti	0.00	0.6000	0.0000	0.0000	0.0000	0.0000	0.0000	0.0000
Equatorial Guinea	0.00	0.6000	1.4697	1.2170	1.5458	1.2802	0.7911	0.6552
Eritrea	0.00	0.6000	0.0000	0.0000	0.0000	0.0000	0.0000	0.0000
Ethiopia	0.00	0.6000	0.0000	0.0000	0.0000	0.0000	0.0000	0.0000
Gabon	0.25	0.6000	2.3482	0.0067	2.4458	0.0557	1.2517	0.0285
Gambia	0.23	0.6000	2.4512	0.1038	2.2143	0.0048	1.1332	0.0024
Ghana	0.00	0.6000	0.0000	0.0000	0.0000	0.0000	0.0000	0.0000
Guinea-bissau	0.03	0.6000	0.9486	1.3969	0.7975	1.0964	0.4081	0.5611
Guinea	0.00	0.6000	0.0000	0.0000	0.0000	0.0000	0.0000	0.0000
Kenya	0.00	0.6000	0.0000	0.0000	0.0000	0.0000	0.0000	0.0000
Lesotho	0.00	0.6000	0.0000	0.0000	0.0000	0.0000	0.0000	0.0000
Liberia	0.51	0.6000	4.03005	0.9974	2.4216	0.0482	1.2393	0.0246
Madagascar	0.17	0.6000	1.6689	0.0107	2.2543	0.0878	1.1537	0.0449
Malawi	0.08	0.6000	0.1950	0.3828	0.4673	0.9171	0.2391	0.4693
Mali	0.27	0.6000	2.2928	0.5049	2.1995	0.0288	1.1256	0.0147
Mauritania	0.13	0.6000	1.3109	0.2583	2.2187	0.0305	1.1354	0.0156
Mozambique	0.11	0.6000	0.1224	0.3430	0.2608	0.7299	0.1335	0.3735
Namibia	0.00	0.6000	0.0000	0.0000	0.0000	0.0000	0.0000	0.0000
Niger	0.17	0.6000	1.6632	0.2075	2.1390	0.0309	1.0947	0.0158
Nigeria	0.14	0.6000	1.1007	0.0144	2.3019	0.1188	1.1780	0.0608
Rwanda	0.00	0.6000	0.0000	0.0000	0.0000	0.0000	0.0000	0.0000
Senegal	0.24	0.6000	2.4818	0.0059	2.1963	0.0486	1.1240	0.0249
Seychelles	0.00	0.6000	0.0000	0.0000	0.0000	0.0000	0.0000	0.0000
Sierra Leone	0.11	0.6000	2.0141	0.0029	2.3958	0.0244	1.2261	0.0124
Somalia	0.00	0.6000	0.0000	0.0000	0.0000	0.0000	0.0000	0.0000
South Africa	0.00	0.6000	0.0000	0.0000	0.0000	0.0000	0.0000	0.0000
South Sudan	0.00	0.6000	0.0000	0.0000	0.0000	0.0000	0.0000	0.0000
Sudan	0.00	0.6000	0.0000	0.0000	0.0000	0.0000	0.0000	0.0000
Tanzania	0.12	0.6000	0.0171	0.1261	0.0411	0.3025	0.0210	0.1548
Uganda	0.00	0.6000	0.0000	0.0000	0.0000	0.0000	0.0000	0.0000
Zambia	010	0.6000	0.9585	0.0666	2.2224	0.0155	1.1374	0.0079
Zimbabwe	0.07	0.6000	1.0946	0.00599	2.2522	0.0491	1.1526	0.0251

**Table 4:** Comparison of the different energy generation costs when FR=99%.

Country	Retail Price at 100 kWh/month	*FR*	CES ($/kWh)		DES ($/kWh)		BDES ($/kWh)	
			Mean	Std	Mean	Std	Mean	Std
Angola	0.04	0.9999	0.1994	0.4465	0.4203	1.5036	0.1290	0.4617
Benin	0.30	0.9999	0.6326	0.7953	2.0352	2.8217	0.6250	0.8665
Botswana	0.10	0.9999	0.2971	0.5454	0.9009	2.3057	0.2766	0.7081
Burkina Faso	0.31	0.9999	1.0526	1.3810	1.9672	2.5577	0.6041	0.7854
Burundi	0.00	0.9999	0.0000	0.0000	0.0000	0.0000	0.0000	0.0000
Cameroon	0.10	0.9999	0.4922	0.7051	1.3447	2.5277	0.4129	0.7762
Central African Republic	0.00	0.9999	0.0000	0.0000	0.0000	0.0000	0.0000	0.0000
Chad	0.18	0.9999	0.1993	0.6057	0.4789	1.4517	0.1470	0.4458
Congo	0.00	0.9999	0.7927	1.2815	1.7594	2.8487	0.5403	0.8748
Congo drc	0.12	0.9999	0.1232	0.3860	0.5102	1.5988	0.1566	0.4909
Co´te d’ivoire	0.27	0.9999	0.6730	1.6236	0.8720	2.0597	0.2677	0.6325
Djibouti	0.00	0.9999	0.0000	0.0000	0.0000	0.0000	0.0000	0.0000
Equatorial Guinea	0.00	0.9999	1.7695	1.4660	4.003	3.3391	1.2295	1.0254
Eritrea	0.00	0.9999	0.0000	0.0000	0.0000	0.0000	0.0000	0.0000
Ethiopia	0.02	0.9999	0.0000	0.0000	0.0000	0.0000	0.0000	0.0000
Gabon	0.25	0.9999	2.8847	0.0547	6.8447	0.4488	2.1019	0.1378
Gambia	0.23	0.9999	3.0637	0.1164	7.2366	0.5049	2.2223	0.1550
Ghana	0.12	0.9999	0.0000	0.0000	0.0000	0.0000	0.0000	0.0000
Guinea-bissau	0.03	0.9999	1.1014	1.5934	2.0505	2.8264	0.6297	0.8680
Guinea	0.00	0.9999	0.0000	0.0000	0.0000	0.0000	0.0000	0.0000
Kenya	0.23	0.9999	0.0000	0.0000	0.0000	0.0000	0.0000	0.0000
Lesotho	0.11	0.9999	0.0000	0.0000	0.0000	0.0000	0.0000	0.0000
Liberia	0.51	0.9999	4.6311	1.0641	7.3504	1.3603	2.2572	0.4177
Madagascar	0.17	0.9999	2.3126	0.1468	7.5331	1.2039	2.3134	0.3697
Malawi	0.08	0.9999	0.2727	0.5354	1.1043	2.1687	0.3391	0.6660
Mali	0.27	0.9999	2.8391	0.5060	6.6791	1.0373	2.0511	0.3185
Mauritania	0.13	0.9999	2.9692	1.7231	7.6160	0.5423	2.3388	0.1665
Mozambique	0.11	0.9999	0.2890	0.5375	0.8074	2.2921	0.2479	0.7039
Namibia	0.00	0.9999	0.0000	0.0000	0.0000	0.0000	0.0000	0.0000
Niger	0.17	0.9999	2.0298	0.2237	5.1456	0.6384	1.5802	0.1960
Nigeria	0.14	0.9999	2.5753	1.6047	6.1936	1.4354	1.9020	0.4408
Rwanda	0.23	0.9999	0.0000	0.0000	0.0000	0.0000	0.0000	0.0000
Senegal	0.24	0.9999	3.0648	0.0908	6.9773	0.7445	2.1427	0.2286
Seychelles	0.00	0.9999	0.0000	0.0000	0.0000	0.0000	0.0000	0.0000
Sierra Leone	0.11	0.9999	2.6199	0.1308	7.3634	1.0733	2.2612	0.3296
Somalia	0.00	0.9999	0.0000	0.0000	0.0000	0.0000	0.0000	0.0000
South Africa	0.00	0.9999	0.0000	0.0000	0.0000	0.0000	0.0000	0.0000
South Sudan	0.00	0.9999	0.0000	0.0000	0.0000	0.0000	0.0000	0.0000
Sudan	0.00	0.9999	0.0000	0.0000	0.0000	0.0000	0.0000	0.0000
Tanzania	0.12	0.9999	0.1243	0.3525	0.1003	0.7374	0.0308	0.2264
Uganda	0.22	0.9999	0.0000	0.0000	0.0000	0.0000	0.0000	0.0000
Zambia	0.10	0.9999	1.9092	1.3817	5.9182	0.6693	1.8174	0.2055
Zimbabwe	0.07	0.9999	2.6770	1.6369	7.0274	0.7195	2.1581	0.2209

**Table 5:** Comparison of the selling and buying cost of different unmet load consumption.

Unmet loads	Selling Cost ($/kWh)	Buying Cost ($/kWh)	Offer Price ($/kWh)	Blockchain Price ($/kWh)
Constant load	2.05	153.86	6.60	0.088
Medium load	2.60	386.23	7.58	0.051
Heavy night load	4.53	226.91	9.84	0.096
Business daily load	8.82	119.94	23.63	0.095

## Abbreviations

BLCOE	Blockchain-based levelized cost of energy
BDES	greenBlockchain-based decentralized energy system
CP	Critical prosumer
CES	Centralized energy system
DSM	Demand side management
DES	Decentralized energy system
DER	Distributed energy resource
FR	Fill rate
LCOE	Levelized cost of energy
NCP	Non-critical prosumer
OM	Operation and maintenance cost
P2P	Peer-to-peer
PV	Photovoltaic
RES	Renewable energy source
SHS	Solar home system
SSA	Sub-Sahara Africa
SG	Smart grid
SL	Service level
WC	Working capital
WCR	Working capital ratio

## References

[1] Ng E.J., El-Shatshat R.A. Multi-microgrid control systems (MMCS). Proceedings of the IEEE PES General Meeting.

[2] Bruck M., Sandborn P., Goudarzi N. (2018). A Levelized Cost of Energy (LCOE) model for wind farms that include Power Purchase Agreements (PPAs). Renew. Energy.

[3] Levin T., Thomas V.M. (2012). Least-cost network evaluation of centralized and decentralized contributions to global electrification. Energy Policy.

[4] Bhatti H.J., Danilovic M. (2018). Making the World More Sustainable: Enabling Localized Energy Generation and Distribution on Decentralized Smart Grid Systems. World J. Eng. Technol..

[5] Patel N., Srinivasan B., Srinivasan R. (2016). Non-intrusive Appliance Load Monitoring for Electrical Energy Systems Simulation and Analysis-A case study in India. Comput. Aided Chem. Eng..

[6] Energypedia Solar Home System. https://energypedia.info/wiki/Solar_Home_Systems_.

[7] International Energy Agency World Energy Outlook. http://www.worldenergyoutlook.org/resources/energydevelopment/energyaccessdatabase/.

[8] Blimpo M.P., Cosgrove-Davies M. (2019). Electricity access in Sub-Saharan Africa: Uptake, reliability, and complementary factors for economic impact. World Bank Publ..

[9] Lowitzsch J. (2019). Financing Renewables While Implementing Energy Efficiency Measures through Consumer Stock Ownership Plans (CSOPs)-The H2020 Project Score.

[10] Meierding E. (2011). Energy security and sub-Saharan Africa. Inst. Dev. Policy Rev. Intern. Politique Dev..

[11] Prasad J., Samikannu R. (2018). Barriers to implementation of smart grids and virtual power plant in sub-saharan region-focus Botswana. Energy Rep..

[12] Chang G., Jones C.A., Roberts J.D., Neary V.S. (2018). A comprehensive evaluation of factors affecting the levelized cost of wave energy conversion projects. Renew. Energy.

[13] Tu Q., Betz R., Mo J., Fan Y., Liu Y. (2019). Achieving grid parity of wind power in China–Present levelized cost of electricity and future evolution. Appl. Energy.

[14] Shea R.P., Ramgolam Y.K. (2019). Applied levelized cost of electricity for energy technologies in a small island developing state: A case study in Mauritius. Renew. Energy.

[15] Hulio Z.H., Jiang W. (2018). Assessment of the apparent performance characterization along with levelized cost of energy of wind power plants considering real data. Energy Explor..

[16] Mendicino L., Menniti D., Pinnarelli A., Sorrentino N. (2019). Corporate power purchase agreement: Formulation of the related levelized cost of energy and its application to a real life case study. Appl. Energy.

[17] Hwang S.H., Kim M.K., Ryu H.S. (2019). Real Levelized Cost of Energy with Indirect Costs and Market Value of Variable Renewables: A Study of the Korean Power Market. Energies.

[18] Gioutsos D.M., Blok K., Velzen L., Moorman S. (2019). Cost-optimal electricity systems with increasing renewable energy penetration for islands across the globe. Appl. Energy.

[19] Lee J.T., Callaway D.S. (2018). The cost of reliability in decentralized solar power systems in sub-Saharan Africa. Nat. Energy.

[20] Bazilian M., Sagar A., Detchon R., Yumkella K. (2010). More heat and light. Energy Policy.

[21] Samuel O., Javaid N., Rabiya K., Muhammad I., Moshen G. Case Study of Direct Communication based Solar Power Systems in Sub-Saharan Africa for Levelled Energy Cost Using Blockchain. Proceedings of the IEEE International Conference on Communications.

[22] Morstyn T., Teytelboym A., McCulloch M.D. (2018). Bilateral contract networks for peer-to-peer energy trading. IEEE Trans. Smart Grid.

[23] Nazari M.H., Costello Z., Feizollahi M.J., Grijalva S., Egerstedt M. (2014). Distributed frequency control of prosumer-based electric energy systems. IEEE Trans. Power Syst..

[24] Morstyn T., Farrell N., Darby S.J., McCulloch M.D. (2018). Using peer-to-peer energy-trading platforms to incentivize prosumers to form federated power plants. Nat. Energy.

[25] Khalid A., Javaid N., Mateen A., Ilahi M., Saba T., Rehman A. (2019). Enhanced time-of-use electricity price rate using game theory. Electronics.

[26] Iria J.P., Soares F.J., Matos M.A. (2018). Trading small prosumers flexibility in the energy and tertiary reserve markets. IEEE Trans. Smart Grid.

[27] Zhang C., Wu J., Zhou Y., Cheng M., Long C. (2018). Peer-to-Peer energy trading in a Microgrid. Appl. Energy.

[28] Tushar W., Saha T.K., Yuen C., Liddell P., Bean R., Poor H.V. (2018). Peer-to-peer energy trading with sustainable user participation: A game theoretic approach. IEEE Access.

[29] Paudel A., Chaudhari K., Long C., Gooi H.B. (2018). Peer-to-Peer Energy Trading in a Prosumer-Based Community Microgrid: A Game-Theoretic Model. IEEE Trans. Ind. Electron..

[30] Tushar W., Saha T.K., Yuen C., Morstyn T., McCulloch M.D., Poor H.V., Wood K.L. (2019). A motivational game-theoretic approach for peer-to-peer energy trading in the smart grid. Appl. Energy.

[31] Livingston D., Sivaram V., Freeman M., Fiege M. (2018). Applying Block chain Technology to Electric Power Systems. Discuss. Pap..

[32] Coinfox European Utilities to Test Blockchain-Based Energy Trading. http://www.coinfox.info/news/7037-european-utilities-to-test-blockchain-based-energy-trading.

[33] Mengelkamp E., Gärttner J., Rock K., Kessler S., Orsini L., Weinhardt C. (2018). Designing microgrid energy markets: A case study: The Brooklyn Microgrid. Appl. Energy.

[34] Münsing E., Mather J., Moura S. Blockchains for decentralized optimization of energy resources in microgrid networks. Proceedings of the IEEE Conference on Control Technology and Applications (CCTA).

[35] Mylrea M., Gourisetti S.N.G. Blockchain for smart grid resilience: Exchanging distributed energy at speed, scale and security. Proceedings of the Resilience Week (RWS).

[36] Khatoon A., Verma P., Southernwood J., Massey B., Corcoran P. (2019). Blockchain in Energy Efficiency: Potential Applications and Benefits. Energies.

[37] Thomason J., Ahmad M., Bronder P., Hoyt E., Pocock S., Bouteloupe J., Donaghy K., Huysman D., Willenberg T., Joakim B. (2018). Blockchain—Powering and Empowering the Poor in Developing Countries. Transforming Climate Finance and Green Investment with Blockchains.

[38] Chelley-Steeley P., Lambertides N., Savva C.S. (2019). Sentiment, order imbalance, and co-movement: An examination of shocks to retail and institutional trading activity. Eur. Financ. Manag..

[39] Wu J., Tran N. (2018). Application of blockchain technology in sustainable energy systems: An overview. Sustainability.

[40] Li W. (2013). Reliability Assessment of Electric Power Systems Using Monte Carlo Methods.

[41] Xiong Z., Zhang Y., Niyato D., Wang P., Han Z. (2018). When mobile blockchain meets edge computing. IEEE Commun. Mag..

[42] Sultana T., Almogren A., Akbar M., Zuair M., Ullah I., Javaid N. (2020). Data Sharing System Integrating Access Control Mechanism using Blockchain-Based Smart Contracts for IoT Devices. Appl. Sci..

[43] Alghamdi T.A., Ali I., Javaid N., Shafiq M. (2019). Secure Service Provisioning Scheme for Lightweight IoT Devices with a Fair Payment System and an Incentive Mechanism based on Blockchain.

[44] Naz M., Al-zahrani F.A., Khalid R., Javaid N., Qamar A.M., Afzal M.K., Shafiq M. (2019). A Secure Data Sharing Platform Using Blockchain and Interplanetary File System. Sustainability.

[45] Rehman M., Javaid N., Awais M., Imran M., Naseer N. Cloud based secure service providing for IoTs using blockchain. Proceedings of the IEEE Global Communications Conference: Communication Information Systems Security.

[46] Samuel O., Javaid N., Awais M., Ahmed Z., Imran M., Guizani M. A Blockchain Model for Fair Data Sharing in Deregulated Smart Grids. Proceedings of the IEEE Global Communications Conference: Communication Information Systems Security.

[47] Wu J., Hu J., Ai X., Zhang Z., Hu H. (2019). Multi-time scale energy management of electric vehicle model-based prosumers by using virtual battery model. Appl. Energy.

[48] Hou J., Wang H., Liu P. (2018). Applying the blockchain technology to promote the development of distributed photovoltaic in China. Int. J. Energy Res..

[49] Aujla G.S., Kumar N., Singh M., Zomaya A.Y. (2019). Energy trading with dynamic pricing for electric vehicles in a smart city environment. J. Parallel Distrib. Comput..

[50] Xu M., Niu J., Lin Y. (2018). An efficient method for fractional nonlinear differential equations by quasi-Newton’s method and simplified reproducing kernel method. Math. Methods Appl. Sci..

[51] Thomas L., Long C., Burnap P., Wu J., Jenkins N. Automation of the supplier role in the GB power system using blockchain-based smart contracts. Proceedings of the 24th International Conference Exhibition on Electricity Distribution (CIRED).

[52] Luthander R., Widén J., Nilsson D., Palm J. (2015). Photovoltaic self-consumption in buildings: A review. Appl. Energy.

[53] Merei G., Moshövel J., Magnor D., Sauer D.U. (2016). Optimization of self-consumption and techno-economic analysis of PV-battery systems in commercial applications. Appl. Energy.

[54] Streatfeild J.E. (2018). Low Electricity Supply in Sub-Saharan Africa: Causes, Implications, and Remedies. J. Int. Commer. Econ..

[55] Business Jargons Types of Incentive Schemes. https://https://businessjargons.com/types-of-incentive-schemes.html/.

[56] Lighting Africa Off-Grid Solar Market Trends Report. https://www.lightingafrica.org/wp-content/uploads/2018/02/2018_Off_Grid_Solar_Market_Trends_Report_Full.pdf.

[57] Ikram A., Su Q., Fiaz M. (2018). Pakistan’s persistent energy crisis and performance of private power producers. Int. J. Bus. Perform. Manag..

[58] Business Dictionary What Is Negative Cash Flow. http://www.businessdictionary.com/definition/negative-cash-flow.html.

[59] National Aeronautics and Space Administration Power Project Data Sets. https://power.larc.nasa.gov/.

[60] Energypedia Burkina Faso Energy Situation. https://energypedia.info/wiki/Burkina_Faso_Energy_Situation.

[61] International Trade Administration Gambia-Energy. https://www.export.gov/article?id=Gambia-Energy.

[62] Surroop D., Raghoo P. (2018). Renewable energy to improve energy situation in African island states. Renew. Sustain. Energy Rev..

[63] Salif S., Sarkodie O.W., Gnamien C.K. Renewable Energy Technology Assessment: Case Study of Senegal, Ghana & Cote d’Ivoire. https://energypedia.info/images/c/c5/Renewable_Energy_Technology_Assessment_Case_Study_of_Senegal_Ghana_and_CC3B4te_dE28099Ivoire.pdf.

[64] United States Agency for International Development Liberia Power Africa Fact Sheet. https://www.usaid.gov/powerafrica/liberia.

[65] Touré A.F., Addouche S.A., Danioko F., Diourté B., Mhamedi A.E. (2019). Hybrid Systems Optimization: Application to Hybrid Systems Photovoltaic Connected to Grid. A Mali Case Study. Sustainability.

[66] Asumadu-Sarkodie S., Owusu P.A. (2017). A multivariate analysis of carbon dioxide emissions, electricity consumption, economic growth, financial development, industrialization, and urbanization in Senegal. Energy Sources Part B Econ. Plan. Policy.

